# Transferrin-Bearing, Zein-Based Hybrid Lipid Nanoparticles for Drug and Gene Delivery to Prostate Cancer Cells

**DOI:** 10.3390/pharmaceutics15112643

**Published:** 2023-11-20

**Authors:** Khadeejah Maeyouf, Intouch Sakpakdeejaroen, Sukrut Somani, Jitkasem Meewan, Hawraa Ali-Jerman, Partha Laskar, Margaret Mullin, Graeme MacKenzie, Rothwelle J. Tate, Christine Dufès

**Affiliations:** 1 Strathclyde Institute of Pharmacy and Biomedical Sciences, University of Strathclyde, Glasgow G4 0RE, UK; khadeejah.maeyouf.2018@uni.strath.ac.uk (K.M.); intouch@tu.ac.th (I.S.); sukrut.somani@outlook.com (S.S.); jitkasem.meewan@strath.ac.uk (J.M.); hawraa-f-r-m-ali@strath.ac.uk (H.A.-J.); plaskar@gitam.edu (P.L.); graeme.mackenzie@strath.ac.uk (G.M.); r.j.tate@strath.ac.uk (R.J.T.); 2 Faculty of Medicine, Thammasat University, Klong Nueng, Klong Luang, Pathumthani 12121, Thailand; 3 Department of Chemistry, School of Science, Gandhi Institute of Technology and Management, Visakhapatnam 530045, Andhra Pradesh, India; 4 Glasgow Imaging Facility, College of Medical, Veterinary and Life Sciences, University of Glasgow, Glasgow G12 8QQ, UK; margaret.mullin@glasgow.ac.uk

**Keywords:** zein-based hybrid lipid nanoparticles, transferrin, tumor targeting, delivery system, cancer therapy, prostate cancer

## Abstract

Gene therapy holds great promise for treating prostate cancer unresponsive to conventional therapies. However, the lack of delivery systems that can transport therapeutic DNA and drugs while targeting tumors without harming healthy tissues presents a significant challenge. This study aimed to explore the potential of novel hybrid lipid nanoparticles, composed of biocompatible zein and conjugated to the cancer-targeting ligand transferrin. These nanoparticles were designed to entrap the anti-cancer drug docetaxel and carry plasmid DNA, with the objective of improving the delivery of therapeutic payloads to prostate cancer cells, thereby enhancing their anti-proliferative efficacy and gene expression levels. These transferrin-bearing, zein-based hybrid lipid nanoparticles efficiently entrapped docetaxel, leading to increased uptake by PC-3 and LNCaP cancer cells and significantly enhancing anti-proliferative efficacy at docetaxel concentrations exceeding 1 µg/mL. Furthermore, they demonstrated proficient DNA condensation, exceeding 80% at polymer–DNA weight ratios of 1500:1 and 2000:1. This resulted in increased gene expression across all tested cell lines, with the highest transfection levels up to 11-fold higher than those observed with controls, in LNCaP cells. These novel transferrin-bearing, zein-based hybrid lipid nanoparticles therefore exhibit promising potential as drug and gene delivery systems for prostate cancer therapy.

## 1. Introduction

Prostate cancer, the second leading cause of cancer-related deaths in men, claims the lives of over 300,000 patients worldwide per year. Its incidence has continued to rise over the last two decades [1,2,3,4]. While treatments like cryoablation, chemotherapy, radiotherapy, and radical prostatectomy can be efficacious against localized tumors, there is still no effective treatment for patients with recurrent or metastatic disease [5]. Consequently, there is an urgent need for new therapeutic approaches to address the needs of these patients.

Among novel experimental strategies, gene therapy holds great promise for the treatment of prostate cancer. However, its practical application is currently hindered by the lack of suitable delivery systems capable of transporting therapeutic DNA and drugs while specifically targeting tumors, without causing adverse effects on healthy tissues [6].

To address this challenge, we hypothesize that the use of hybrid lipid nanoparticles composed of biocompatible and biodegradable zein and conjugated to transferrin (whose receptors are overexpressed on prostate cancer cells [7,8,9]) would be able to entrap the hydrophobic anti-cancer drug docetaxel and carry plasmid DNA. It is expected that these nanoparticles would enhance the delivery of therapeutic payloads to prostate cancer cells, consequently increasing their anti-proliferative efficacy and gene expression levels.

Zein, a hydrophobic and biodegradable protein extracted from corn, has received approval from the US Food and Drug Administration as generally regarded as safe (GRAS) for various applications in food, pharmaceutical, and biomedical industries [10,11,12,13]. Due to its amphiphilic molecular structure, zein exhibits solubility in 50–90% (*v*/*v*) aqueous ethanol, but its composition, comprising more than 50% lipophilic amino acids, renders it insoluble in water. Additionally, its high glutamine content makes it insoluble in absolute alcohol [14]. By taking advantage of its different solubilities in ethanol and water, zein has demonstrated significant potential as a carrier system for the delivery of nutraceuticals, drugs, and DNA [15,16,17,18]. Furthermore, the modification of zein through conjugation with poly(ethylene glycol) (PEG) has been shown to introduce a steric shielding of the carrier system [19], therefore decreasing any potential opsonization and providing a sustained release of the entrapped drug [20,21,22,23].

The drug selected to be entrapped within the zein-based hybrid lipid nanoparticles, docetaxel, is a semi-synthetic analogue of paclitaxel used as an anti-neoplastic therapy for the treatment of prostate tumors. It was approved by both the FDA and the European Medicines Agency in 2004 as afirst-line treatment for advanced prostate cancer [24]. Due to its bulky polycyclic structure [25], docetaxel is insoluble in water. It exerts its therapeutic effect by hyper-stabilizing the protein structure of microtubules, inhibiting their disassembly through binding to the beta-tubulin subunit. This leads to the persistence of microtubule structure, a loss of cytoskeleton flexibility, cell cycle arrest, and ultimately, cell death. Docetaxel is employed as a first-line treatment option for prostate cancer, either as a single agent or in combination with estramustine for androgen-independent prostate cancer. However, due to its high lipophilicity, which adversely impacts its pharmacokinetic profile, commercially available docetaxel formulations incorporate the surfactant Tween^®^ 80 to enhance its solubility, physicochemical properties, and its cell membrane permeability [26]. Furthermore, docetaxel is associated with various adverse effects, including high toxicity, low sensitivity, and the potential development of resistance, thereby limiting its therapeutic efficacy [24,27]. It is therefore important to entrap this drug within a delivery system that would deliver it specifically to its site of action.

The objectives of this study were therefore to synthesize and characterize transferrin-bearing, zein-based hybrid lipid nanoparticles, to evaluate their ability to entrap docetaxel and complex a plasmid DNA, and to assess their cellular uptake, transfection, and anti-proliferative efficacy on prostate cancer cells.

## 2. Materials and Methods

### 2.1. Reagents and Cell Lines

Yellow zein, human holo-transferrin (Tf), cupric sulphate (pentahydrate), 2-iminothiolane hydrochloride (Traut’s reagent), 2-nitrophenyl-β-D-galactopyranoside (ONPG), docetaxel *purum* (≥97.0%), Vivaspin^®^ 6 centrifuge tubes with a molecular weight cut-off (MWCO) of 5000 and 100,000 Daltons, and all the chemicals not mentioned below were obtained from Sigma-Aldrich (Poole, UK). 1,2-distearoyl-sn-glycero-3-phosphoethanolamine-N- [maleimide (polyethylene glycol)-2000] (DSPE-PEG2K-MAL) was purchased from Jenkem Technology (Plano, TX, USA). 1,2-distearoyl-sn-glycero-3-phosphoethanolamine-N-(carbonyl methoxypolyethylene-glycol 2000), sodium salt (DSPE-PEG2K) was obtained from NOF Corporation (Tokyo, Japan). The expression plasmid encoding β-galactosidase (pCMVsport β-galactosidase) was obtained from Invitrogen (Paisley, UK) and was purified using an Endotoxin-free Giga Plasmid Kit (Qiagen, Hilden, Germany). Bioware^®^ androgen-irresponsive PC-3M-luc-C6 human prostate adenocarcinoma that expresses the firefly luciferase was purchased from Caliper Life Sciences (Hopkinton, MA, USA), while the androgen-irresponsive DU145 and androgen-sensitive LNCaP prostate cancer cell lines came from the European Collection of Cell Cultures (Salisbury, UK). Roswell Park Memorial Institute 1640 (RPMI) cell culture medium, Quant-iT^®^ PicoGreen^®^ dsDNA reagent, fetal bovine serum (FBS), sodium pyruvate, L-glutamine, N-2-hydroxyethylpiperazine-N-2-ethane sulfonic acid (HEPES), penicillin-streptomycin, TrypLE^®^ Express, and Tubulin Tracker^®^ Green (Oregon Green^®^ 488 Taxol, bis-acetate) were purchased from Life Technologies (Paisley, UK). Passive lysis 5× buffer was obtained from Promega (Southampton, UK). Vectashield^®^ mounting medium containing 4′,6-diamidino-2-phenylindole (DAPI) came from Vector Laboratories (Peterborough, UK).

### 2.2. Evaluation of the Concentration of Ethanol Needed to Dissolve Zein

To assess the solubility of zein in ethanol, 2 mL of ethanol (50, 60, 70, 80, and 90%) was added to 20 mg of zein and thorough mixing ensued to yield a transparent, yellow solution. The subsequent nanoparticle preparation was performed by employing the specific ethanol concentration at which zein demonstrated complete dissolution.

### 2.3. Preparation and Optimization of Zein-Based Hybrid Lipid Nanoparticles Entrapping Coumarin

#### 2.3.1. Comparison of One-Step Nanoprecipitation and Coacervation Methods

Zein-based hybrid lipid nanoparticles entrapping coumarin-6 were prepared by using both one-step nanoprecipitation and coacervation methods.

Initially, the nanoparticles were prepared through the nanoprecipitation method, using the ethanol concentration (80%) that enabled complete zein dissolution.

The other experimental parameters were first fixed: the weight ratio of lipid 1,2-distearoyl-sn-glycero-3-phosphoethanolamine-N-[maleimide (polyethylene glycol)-2000] (DSPE-PEG2K-MAL) to zein at 1:5 (*w*/*w*), the volume ratio of water to ethanol at 2:1 (*v*/*v*), the zein concentration of 10 mg/mL in the organic solvent, and the theoretical drug loading at 0.5% of zein weight.

To initiate the process, DSPE-PEG2K-MAL (6 mg) was dispersed in 6 mL of Milli-Q ultrapure water (15.0 MΩ·cm) at 65 °C with continuous shaking at 700 rpm for 1 h. Subsequently, a solution of coumarin-6 (150 µg in 75 µL DMSO) was added to the zein solution (30 mg in 3 mL ethanol (80%)). The resulting mixture was then introduced dropwise into the lipid phase and stirred for 15 min at 25 °C, allowing for the formation of homogenous nanoparticles. The resulting nanoparticles were collected by centrifugation conducted at 8000 rpm (14,000× *g*) for 20 min at 20 °C, using a Hermle^®^ Z323K centrifuge (Wehingen, Germany). The collected nanoparticles underwent a washing step with 1 mL of ultrapure water to remove any unentrapped coumarin-6. After washing, the nanoparticles were centrifuged once more under the same conditions. Subsequently, the nanoparticles were resuspended in 1 mL of ultrapure water and stored at 4 °C. Control zein-based hybrid lipid nanoparticles entrapping coumarin-6 were prepared using an identical protocol, with the exception that DSPE-PEG2K-MAL was replaced with DSPE-PEG2K as the lipid component.

In the coacervation method, aqueous and organic solutions were prepared following the same procedures as outlined in the nanoprecipitation method. However, the sequence of addition was reversed. Specifically, the aqueous solution (consisting of lipid dispersed in water) was added dropwise into the organic solution (comprising zein dissolved in ethanol). This addition was carried out under identical conditions as those employed in the nanoprecipitation method.

#### 2.3.2. Variation in Water–Ethanol Volume Ratios

Zein-based hybrid lipid nanoparticles entrapping coumarin-6 were prepared via the nanoprecipitation method. Various volume ratios of water–ethanol (2:1, 3:1, 4:1, and 5:1) corresponding to 6, 9, 12, 15 mL of ultrapure water were used. Meanwhile, the lipid–zein weight ratio was maintained at 1:5, the zein concentration in the organic solution was set at 10 mg/mL, and the loading of coumarin-6 was fixed at 0.5% of the zein weight. DSPE-PEG2K-MAL (6 mg) was dispersed through stirring at 65 °C for 1 h in 6, 9, 12, and 15 mL of ultrapure water. Subsequently, a solution of coumarin-6 (150 µg in 75 µL DMSO) was added to the zein solution (30 mg in 3 mL ethanol (80%)). The nanoparticles were then generated, collected, and stored as described above.

#### 2.3.3. Use of Probe Sonication

The nanoparticles were prepared as described above, with the inclusion of an extra step involving probe sonication (sonication for 3 × 2 min at 21% amplitude using a Vibra-Cell^®^ probe sonicator (Sonics, Newtown, CT, USA)). Following this, the nanoparticles were gathered through centrifugation.

#### 2.3.4. Addition of One-Hour Incubation before Centrifugation

The nanoparticles were prepared as described above, with the addition of a one-hour incubation period following the dropwise addition of the organic solution to the aqueous solution at 25 °C, prior to the centrifugation step.

#### 2.3.5. Variation in Lipid–Zein Weight Ratios

Three distinct weight ratios of lipid–zein were used: 1:4, 1:5, and 1:6. The water–ethanol volume ratio was held at 2:1, with a constant zein amount of 30 mg and a zein concentration of 10 mg/mL in the organic solvent. Various lipid amounts (5, 6, and 7.5 mg) were dispersed in 6 mL of ultrapure water. The zein solution was prepared and added to the lipid dispersion as described above, after which probe sonication was carried out for three cycles of two minutes each. Subsequently, the nanoparticles were gathered via centrifugation, in accordance with the method described above.

#### 2.3.6. Variation in the Amount of Zein

Nanoparticles were prepared using three amounts of zein: 30, 40, and 50 mg. The drug loading remained constant at 0.5% of the zein weight, with the zein concentration in the organic solvent set at 10 mg/mL. The water–ethanol ratio was maintained at 2:1, while the lipid–zein ratio was fixed at 1:5. Lipids (6, 8, and 10 mg) were dispersed in 6, 8, and 10 mL of ultrapure water as described above. For the incorporation of coumarin-6, solutions containing 150 µg in 75, 100, and 125 µL of DMSO were introduced to zein solutions containing 30, 40, and 50 mg of zein in 3, 4, and 5 mL of 80% ethanol, respectively. The zein solutions were then added dropwise to the aqueous solutions as described above.

#### 2.3.7. Variation in Drug Amount Related to Zein Weight

Nanoparticle formulations were prepared by using two distinct percentages of coumarin-6 relative to the zein weight. Specifically, coumarin-6 was loaded at two ratios (0.1 and 0.5% of the zein weight), while maintaining a fixed zein amount of 40 mg. The lipid to zein weight ratio was set at 1:5, and the volume ratio of water to ethanol was maintained at 2:1. Lipids (8 mg) were dispersed in 8 mL of ultrapure water through stirring for a duration of 1 h within the temperature range of 60–65 °C. Subsequently, 40 µg of coumarin-6 in 20 µL DMSO (for a 0.1% loading of zein weight) and 200 µg of coumarin-6 in 100 µL DMSO (for a 0.5% loading of zein weight) was added to 40 mg of zein dissolved in 4 mL of 80% ethanol. Zein solutions were subsequently added dropwise into the lipid solution as described above.

The amount of coumarin-6 entrapped within the nanoparticles was measured through spectrofluorimetry, using an Agilent Varian Cary Eclipse^®^ spectrofluorometer (Agilent Technologies, Santa Clara, CA, USA). The excitation wavelength (λ_exc_) was set at 463 nm, while the emission wavelength (λ_em_) was set at 510 nm, with a slit width of 5 nm. The results were expressed as a percentage of entrapment efficiency.

### 2.4. Preparation of Transferrin-Bearing Zein-Based Hybrid Lipid Nanoparticles Encapsulating Docetaxel

Zein nanoparticles entrapping docetaxel were prepared through the nanoprecipitation method as refined based on the optimization using coumarin-6. Briefly, DSPE-PEG2K-MAL (8 mg) was dispersed in 8 mL of ultrapure water by shaking at 700 rpm at 60–65 °C for a duration of 1 h. A solution of docetaxel (200 µg in DMSO) was added to the zein solution (40 mg in 4 mL of 80% ethanol). The organic phase, consisting of zein dissolved in ethanol, was added dropwise into the aqueous phase, where lipid was dispersed in water, and mixed through stirring at 25 °C for 15 min. Following this, the mixture underwent an additional 15 min of stirring to enhance uniformity before undergoing probe sonication for three cycles of two minutes each. The nanoparticles were then subjected to centrifugation at 14,000× *g* for a duration of 20 min at 20 °C, using an Avanti^®^ J-E centrifuge (Beckman Coulter, London, UK). To remove unentrapped docetaxel, the nanoparticles were subjected to a wash step using 2 mL of ultrapure water, followed by another round of centrifugation under identical conditions. Ultimately, the nanoparticles had their final volume adjusted to 1 mL with ultrapure water and were stored at 4 °C. The amount of docetaxel in the nanoparticles was determined through spectrophotometry (λ_max_: 312 nm), using an Agilent Varian Cary^®^ 50 UV-Vis spectrophotometer (Agilent Technologies, Santa Clara, CA, USA). The results were expressed as percentage of entrapment efficiency.

Transferrin was conjugated to the nanoparticles through the thiol–maleimide ‘click’ reaction method described by Hermanson [28]. Initially, 10 mg of transferrin dissolved in 1 mL of 50 mM sodium phosphate buffer containing 0.15 M sodium chloride (pH 8) was reacted with a 10-fold molar excess of Traut’s reagent (2-iminothiolane hydrochloride) (85 µL of Traut’s reagent, 2 mg/mL in deionized water) under moderate stirring at 25 °C for a duration of 2 h. The resultant thiolated Tf was then isolated from the unreacted reagent through centrifugation at 9500 rpm (10,500× *g*) for 15 min at 20 °C (Hermle^®^ Z323K centrifuge, Wehingen, Germany), using Vivaspin^®^ 6 centrifuge tubes with a molecular weight cut-off of 5000 Daltons (Sartorius Ltd., Epsom, UK). It was immediately conjugated to 1 mL of nanoparticles prepared as described above while under continuous stirring at 25 °C for 1 h. Any residual unreacted Tf was subsequently removed from the hybrid lipid nanoparticles by centrifugation at 7500 rpm (6600× *g*) for 15 min at 20 °C, using Vivaspin^®^ 6 centrifuge tubes with a molecular weight cut-off of 100,000 Daltons.

Parallelly, control nanoparticles underwent purification through the same centrifugation method as described above, using Vivaspin^®^ 6 centrifuge tubes with a molecular weight cut-off of 5000 Daltons. Post purification, their final volume was adjusted to 1 mL using ultrapure water and they were then stored at 4 °C.

The amount of transferrin conjugated to the nanoparticles was determined by the Lowry assay [29], using a previously established protocol [30].

The morphology of transferrin-bearing and control zein-based hybrid lipid nanoparticles was visualized through transmission electron microscopy. Initially, formvar/carbon-coated copper grids (400 mesh) were subjected to glow discharge. Subsequently, a 3–5 μL droplet of each sample, previously diluted to a 1:10 ratio with deionized water, was deposited onto the hydrophilic support film and left to air-dry overnight. The dried samples were then subjected to imaging using a Jeol JEM-1200EX^®^ transmission electron microscope (Jeol, Peabody, MA, USA), operating at an accelerating voltage of 80 kV and equipped with a Gatan 794 MultiScan^®^ camera (Gatan, Pleasanton, CA, USA).

The size and zeta potential of Tf-bearing and control zein-based hybrid lipid nanoparticles entrapping docetaxel were measured using photon correlation spectroscopy and laser Doppler electrophoresis, using a Zetasizer Nano-ZS^®^ (Malvern Instruments Ltd., Malvern, UK). All samples were appropriately diluted to a 1:50 ratio in ultrapure water. The diluted samples were then brought to a final volume of 1 mL to fall within the analytical measurement range before being transferred into disposable cuvettes and folded capillary cells for size and zeta potential measurement, respectively. The experiment was performed in quadruplicate.

### 2.5. Stability of the Formulations

The stability of Tf-conjugated and control zein-based hybrid lipid nanoparticles entrapping docetaxel, along with the blank nanoparticles, was assessed through a systematic process. All samples were securely sealed in tightly closed tubes, shielded from light, and then stored under controlled conditions at 4 °C over a span of 4 weeks. To monitor the stability, the size and zeta potential of the nanoparticles were, respectively, measured using the Zetasizer Nano-ZS^®^, as described above. Measurements were conducted on Days 0, 7, 14, 21, and 28 to gauge any changes over time. The amount of docetaxel retained within the formulations was quantified via spectrophotometry.

### 2.6. Drug Release

The release profile of docetaxel was assessed using a dialysis technique at various pH conditions (5.5, 6.5, and 7.4), simulating the acidic endosomal pH of cancer cells, the extracellular environment pH of tumors, and the physiological pH found in normal tissue and blood, respectively. To do so, docetaxel either formulated as Tf-bearing, control zein-based hybrid lipid nanoparticles, or in solution (500 µg of docetaxel in 2.5 mL distilled water) was placed into a SnakeSkin^®^ dialysis tube with a molecular weight cut-off of 7000 Da (ThermoFisher Scientific, Waltham, MA, USA) and was dialyzed against 100 mL of phosphate buffer (pHs 5.5, 6.5, and 7.4) at 37 °C under gentle stirring. Throughout the experiment, aliquots of 1 mL of the dialysate were withdrawn in triplicate at specific time intervals (30 min, then every hour for the initial six hours (1, 2, 3, 4, 5, and 6 h), then every 2 h for the next 6 h (8, 10, and 12 h), and 24 h. For each sample, the withdrawn volume was replenished with 3 mL of fresh buffer, maintaining a consistent buffer volume of 100 mL. The amount of docetaxel in the formulations was quantified through spectrophotometry using an Agilent Varian Cary^®^ 50 UV-Vis spectrophotometer set at an absorption wavelength of 312 nm. The results were expressed as a percentage cumulative drug release.

### 2.7. In Vitro Studies

#### 2.7.1. Cellular Uptake

Flow cytometry was employed as a method for quantifying the fluorescence intensity of docetaxel that was internalized by the cancer cells. To this end, PC3-Luc, DU145, and LNCaP cells were initially seeded at a density of 1 × 10^6^ cells/well in 6-well plates and cultured at a temperature of 37 °C for a period of 24 h. Following the removal of the culture medium, the cells were treated with fluorescein-labelled docetaxel (12 μg/well), which was either entrapped within Tf-bearing or control zein-based hybrid lipid nanoparticles or in solution, for an additional 15 h at a temperature of 37 °C. Following the incubation period, the cells were subjected to thorough washing, involving 3 washes with cold PBS (2 mL). Subsequently, they were detached from the plates using TrypLE^®^ Express (250 µL). The trypsinization reaction was then stopped by adding 500 µL of fetal bovine serum (FBS) (1% in PBS) to the cell suspension. The mean fluorescence intensity (MFI) of the drug taken up by the cells was measured through using Attune^®^ NxT Acoustic Focusing cytometer (Thermo Fisher Scientific, Waltham, MA, USA), with Attune^®^ NxT Auto Sampler software version 3.1.2 (Thermo Fisher Scientific, Waltham, MA, USA), using a FITC filter (Exc_max_: 494 nm/Em_max_: 520 nm). Thirty thousand cells (gated events) were counted and analyzed for each sample.

The cellular uptake of fluorescein-labelled docetaxel entrapped within transferrin-bearing and control zein-based hybrid lipid nanoparticles, or in solution, was also qualitatively assessed through confocal microscopy. PC3-Luc, DU145, and LNCaP cells were initially seeded at a density of 2 × 10^5^ cells per well on coverslips in 6-well plates. Subsequently, the cells were incubated for a period of 24 h at a temperature of 37 °C, in a humid atmosphere containing 5% CO_2_. The culture medium was then removed, and the cells were exposed to fluorescein-labelled docetaxel (12 μg/well), formulated either as Tf-bearing or control zein-based hybrid lipid nanoparticles, or as a solution, for 15 h. Following incubation with the respective treatments, the cells were washed three times with 3 mL of phosphate-buffered saline (PBS), before being fixed with 2 mL methanol for a duration of 10 min at a temperature of 20 °C. After staining the cell nuclei with Vectashield^®^ mounting medium containing 4′,6-diamidino-2-phenylindole (DAPI) for 2 h, the imaging of cells was executed using a Leica TCS SP5^®^ confocal microscope (Wetzlar, Germany). DAPI (which stained the cell nuclei) was excited using a 405 nm laser line, with an emission bandwidth ranging from 400 to 480 nm. On the other hand, fluorescein-labeled docetaxel was excited using a 494 nm laser line, with an emission bandwidth spanning from 510 to 530 nm.

#### 2.7.2. Mechanisms of Cellular Uptake

To determine the underlying mechanisms governing the cellular uptake of docetaxel, PC3-Luc cells were seeded in six-well plates at a density of 1 × 10^6^ cells/well and grown for 24 h. They were then pre-treated with the endocytosis inhibitors transferrin (50 µM), chlorpromazine (20 µg/mL), filipin (5 µg/mL), colchicine (40 µg/mL), and poly-L-lysine (40 µg/mL) at 37 °C for 30 min. Afterwards, the treatments were replaced with co-incubation of fluorescein-labelled docetaxel (12 µg per well) entrapped in Tf-bearing nanoparticles and each inhibitor at identical concentrations (except chlorpromazine, which was added at a concentration of 5 µg/mL) for an additional 15 h incubation at 37 °C. The cells then underwent processing for flow cytometry and confocal analysis as described above.

#### 2.7.3. Anti-Proliferative Efficacy

The anti-proliferative efficacy of docetaxel entrapped in targeted and control zein-based hybrid lipid nanoparticles as well as in solution, was assessed through a MTT assay on PC3-Luc, DU145, and LNCaP prostate cancer cells. To do so, cells were seeded at a density of 2000 cells/well in 96-well plates and incubated for a period of 72 h at a temperature of 37 °C, in a humid atmosphere containing 5% CO_2_. Afterward, the cells were treated with various concentrations of docetaxel (ranging from 0.00001 to 30 µg/mL), either entrapped in Tf-bearing or control zein-based hybrid lipid nanoparticles or in solution. After 72 h treatment, 50 µL of MTT solution (0.5% *w*/*v* in PBS) was added to each well and incubated with the cells for 4 h. The solution was then replaced with 200 µL of dimethyl sulfoxide (DMSO) to dissolve the purple formazan product generated by viable cells. Triton X-100 (0.1% *w*/*v* in PBS) and fresh medium were used as positive and negative control, respectively. Three independent experiments for each formulation, with *n* = 5 for each concentration level, were conducted. The optical density of the formazan solution was measured at an absorbance at 570 nm using a Multiskan Ascent^®^ microplate reader (Thermo Labsystems, Beverly, MA, USA). Percentage absorbance values were used to construct dose–response curves. These curves were fitted to determine the IC_50_ values, which represent the concentration of docetaxel that results in a 50% reduction in cell viability.

### 2.8. Evaluation of the Potential Use of Tf-Bearing Zein-Based Hybrid Lipid Nanoparticles for Gene Therapy

#### 2.8.1. Evaluation of DNA Condensation

The ability of Tf-bearing and control zein-based hybrid lipid nanoparticles to complex DNA was assessed through a PicoGreen^®^ assay. PicoGreen^®^ solution was prepared by following the protocol provided by the supplier. PicoGreen^®^ reagent was diluted 200-fold in Tris-EDTA (TE) buffer (10 mM Tris, 1 mM EDTA, adjusted to pH 7.5) on the day of the experiment, in a plastic container to shield it from light. Nanoparticles formulated as Tf-bearing or control zein-based hybrid lipid nanoparticles were prepared as described above. HCL (0.1 M) was added to the nanoparticles to make them positively charged (zeta potentials of 20 and 26 mV for Tf-bearing and control nanoparticles, respectively, at a pH~4). Briefly, 1 mL of PicoGreen^®^ solution was added to 1 mL of nanoparticles–DNA complex at various polymer–DNA weight ratios (2000:1, 1500:1, 1000:1, 500:1, 250:1, 100:1, 50:1, 20:1, 10:1, 5:1, 2:1, 1:1, 0.5:1). The intensity of PicoGreen^®^ fluorescence was immediately measured by spectrofluorimetry (λ_exc_: 480 nm, λ_em_: 520 nm). The DNA concentration was maintained at a consistent level of 10 µg/mL throughout the experiment. Fluorescence measurements were conducted at multiple time points (0, 30 min, 1 h, 2 h, 4 h, 6 h, 24 h) with *n* = 4 to monitor the interaction between the nanoparticles and DNA.

DNA condensation was also assessed using a gel retardation assay. Tf-bearing and control zein-based hybrid lipid nanoparticles complexed with DNA were prepared as outlined above. After mixing with the loading buffer (2 µL), the resulting samples (15 µL) were loaded onto a 1× Tris-Borate-EDTA (TBE) (89 mM Tris base, 89 mM boric acid, 2 mM Na_2_-EDTA, pH 8.3) buffered 0.8% (*w*/*v*) agarose gel containing ethidium bromide (0.4 µg/mL), with 1×TBE as a running buffer and Bioline HyperLadder^®^ 1 kb as a DNA size marker. Electrophoresis was conducted at a constant voltage of 50 V for a duration of 1 h. The gel was then visualized under UV light, allowing for the observation of the migration of DNA bands.

#### 2.8.2. Measurement of the Size and Zeta Potential of the Complexes

The size and zeta potential of Tf-bearing and control zein-based hybrid lipid nanoparticles–DNA complexes in TE buffer were measured for various polymers–DNA weight ratios ranging from 50:1 to 2000:1, by photon correlation spectroscopy and laser Doppler electrophoresis, using a Malvern Zetasizer Nano-ZS^®^ (Malvern Instruments Ltd., Malvern, UK). The DNA concentration was maintained at a constant level of 10 µg/mL throughout the experiment. The experiment was conducted in quadruplicate.

#### 2.8.3. Gene Expression

A transfection assay was performed to assess the expression of the plasmid DNA encoding β-galactosidase complexed to Tf-bearing and control zein-based hybrid lipid nanoparticles, in PC3-Luc, DU145, and LNCaP prostate cancer cells. The cells were seeded in quintuplicate at a density of 2000 cells/well in 96-well plates and incubated for 72 h at 37 °C in a humid atmosphere with 5% CO_2_. They were then treated with Tf-bearing nanoparticles complexed to plasmid DNA, at six polymers–DNA weight ratios (2000:1, 1500:1, 1000:1, 500:1, 250:1, 100:1). Three independent experiments were conducted. Polyethylenimine (PEI) and generation 3-diaminobutyric polypropylenimine (DAB) dendrimer complexed to DNA (at a polymer–DNA weight ratio of 5:1 for both complexes) were used as positive controls, while naked DNA served as a negative control. The plasmid DNA concentration was maintained at a consistent level of 1 µg per well throughout the entire experiment. After 72 h treatment, the cells were lysed with 1× passive lysis buffer (PLB) (50 μL/well) for 20 min at 37 °C. The cell lysates were then analyzed for β-galactosidase expression by adding 50 µL of 2× assay buffer (sodium phosphate buffer 200 mM; magnesium chloride 0.5 M; β-mercaptoethanol 50 mM; pH 7.3) containing ONPG solution (1.33 mg/mL in 2× assay buffer) to each well containing the lysates. The plates were then incubated for 2 h in the dark at 37 °C before reading the absorbance of each well at 405 nm using a Multiskan Ascent^®^ microplate reader (Thermo Labsystems, Beverly, MA, USA).

### 2.9. Statistical Analysis

The results were expressed as means ± standard error of the mean (S.E.M). Statistical significance was assessed by one-way analysis of variance (ANOVA), and Tukey’s multiple comparison post-test (Minitab^®^ software version 20.0, State College, PE, USA). Differences were considered statistically significant for *p* values lower than 0.05.

## 3. Results

### 3.1. Evaluation of the Concentration of Ethanol Needed to Dissolve Zein

Zein exhibited complete solubility in ethanol concentrations of 70%, 80%, and 90%, resulting in a distinct, pale-yellow solution. Conversely, its solubility was reduced in 50% and 60% ethanol solutions. Zein remained insoluble in absolute ethanol. Based on this finding, the experiments in this study were then conducted by utilizing zein that had been dissolved in ethanol solutions of 70%, 80%, or 90%. This approach aligns with the findings of Paraman and Lamsal [31], as well as Shukla and Cheryan [10], who previously reported the solubility of α-zein in aqueous ethanol concentrations ranging from 60% to 95%.

### 3.2. Preparation and Optimization of Zein-Based Hybrid Lipid Nanoparticles Entrapping Coumarin-6

#### 3.2.1. Comparison of One-Step Nanoprecipitation and Coacervation Methods

Zein-based hybrid lipid nanoparticles were successfully prepared using the nanoprecipitation method with various concentrations of ethanol. They exhibited sizes of 140.3 ± 0.8 nm, 146.6 ± 0.9 nm, and 160.5 ± 1.2 nm for formulations prepared with 70%, 80%, and 90% ethanol in the organic phase, respectively (Table 1). The polydispersity index revealed a narrow size distribution across all these formulations, with values consistently below 0.2 (0.06 ± 0.02, 0.08 ± 0.01, and 0.07 ± 0.01 for the nanoparticles prepared with 70%, 80% and 90% ethanol, respectively). The nanoparticles exhibited a negative charge, with zeta potential values of −29.5 ± 0.5 mV, −30.4 ± 0.8 mV, and −30.5 ± 1.0 mV when prepared using 70%, 80%, and 90% ethanol samples, respectively. These findings collectively indicated robust formulation stability, with a combination of a slightly lower zeta potential and size lower than 150 nm for the formulation prepared with 80% ethanol in the organic phase. Consequently, an ethanol concentration of 80% was selected for nanoparticle preparation.

Hybrid lipid nanoparticles entrapping coumarin-6 as a fluorescent hydrophobic drug model were effectively prepared via both nanoprecipitation and coacervation methods. Through the nanoprecipitation approach, they exhibited a small size (205.7 ± 9.4 nm), coupled with a narrow size distribution (with a PDI of 0.35 ± 0.06) and an inherent negative charge (zeta potential of −24.6 ± 0.8 mV). Impressively, they were able to entrap 74.8 ± 1.1% (equivalent to 112.2 μg) of coumarin-6 (Table 1).

The nanoparticles generated via the coacervation method entrapped a non-statistically different percentage of coumarin-6 (76.4 ± 2.8%, corresponding to 114.6 μg) and displayed a slightly higher negative zeta potential (−21.2 ± 0.5 mV), but exhibited a substantially larger size (572.6 ± 37.5 nm), higher than the cut-off size (400 nm) crucial for extravasation into tumor tissue [32,33]. As a result, the nanoprecipitation method was selected for subsequent optimization.

#### 3.2.2. Variation in Water–Ethanol Volume Ratios

Zein-based hybrid lipid nanoparticles entrapping coumarin-6 were prepared using four distinct water–ethanol volume ratios (2:1, 3:1, 4:1, and 5:1) (Table 1). Their size was relatively close when using water–ethanol ratios of 2:1 and 3:1 (224.3 ± 28.0 nm and 217.1 ± 69.2 nm, respectively), slightly smaller at a ratio of 5:1 (178.9 ± 24.2 nm), but much larger (408.0 ± 73.1 nm) at a ratio of 4:1. Their PDI values exhibited an upward trend as the water–ethanol volume ratio increased from 2:1 to 4:1 (0.25 ± 0.06, 0.40 ± 0.06, and 0.67 ± 0.07 from 2:1 to 4:1, respectively). Their zeta potentials spanned a range from −21 to −23 mV (−21.8 ± 1.0 mV, −23.2 ± 0.8 mV, −23.1 ± 1.2 mV, and −21.6 ± 0.9 mV for the water–ethanol volume ratios 2:1 to 5:1, respectively). Conversely, the percentage of drug entrapment in the nanoparticles widely differed. Optimal entrapment (94.1 ± 2.7%) was achieved at a 2:1 ratio. Contrastingly, the three other ratios yielded notably diminished drug entrapment (51.9 ± 0.9%, 37.7 ± 3.0%, and 32.0 ± 1.6% for ratios 3:1 to 5:1, respectively). A water–ethanol volume ratio of 2:1 was thus selected for further studies.

#### 3.2.3. Use of Probe Sonication

Nanoparticles prepared through probe sonication showcased a size that was non-statistically different when compared to those prepared without probe sonication (236.4 ± 2.0 nm) (Table 1). Their negative zeta potential remained similar (−23.5 ± 3.4 mV). However, their size distribution was narrower (PDI of 0.14 ± 0.05). The subsequent nanoparticle preparation therefore included probe sonication in further experiments.

#### 3.2.4. Addition of One-Hour Incubation before Centrifugation

Incorporating a one-hour incubation period prior to centrifugation yielded nanoparticles characterized by an increased size (260.2 ± 3.1 nm), with a marginally broader size distribution (with a PDI of 0.38 ± 0.07) (Table 1). Their zeta potential exhibited no statistically significant variation (−20.9 ± 1.5 mV). Consequently, this supplementary step was subsequently omitted from the nanoparticle preparation process in subsequent experiments.

#### 3.2.5. Variation in Lipid–Zein Weight Ratios

Three lipid–zein weight ratios (1:4, 1:5, and 1:6) were employed for the formulation of zein-based hybrid lipid nanoparticles entrapping coumarin-6 (Table 1). The nanoparticle sizes exhibited similarity (231.1 ± 3.4 nm, 236.4 ± 2.0 nm, and 224.8 ± 7.9 nm for 1:4, 1:5, and 1:6 ratios, respectively), with low PDI values (0.17 ± 0.02, 0.14 ± 0.05, and 0.15 ± 0.03 for 1:4, 1:5, and 1:6 ratios, respectively). Their zeta potentials demonstrated a negative charge (−17.2 ± 0.7 mV, −23.5 ± 3.4 mV, and −20.1 ± 0.7 mV for ratios 1:4 to 1:6, respectively), with the highest level of stability observed at a 1:5 ratio. Drawing from these findings, the 1:5 ratio was selected for nanoparticle preparation in subsequent experiments.

#### 3.2.6. Variation in the Amount of Zein

Three amounts of zein (30, 40, and 50 mg) were used in nanoparticle preparation. The resultant nanoparticles consistently displayed sizes below 240 nm, with the smallest size achieved utilizing 40 mg of zein (236.4 ± 2.0 nm, 215.9 ± 6.8 nm, and 225.3 ± 6.2 nm for 30, 40, and 50 mg of zein, respectively) (Table 1). The nanoparticles showcased a very narrow size distribution, reflected by PDI values below 0.2 (0.14 ± 0.05, 0.16 ± 0.04, and 0.15 ± 0.01 when using 30, 40, and 50 mg of zein, respectively). Their zeta potential increased with increasing amounts of zein (ranging from −23.5 ± 3.4 mV to −16.6 ± 0.0 mV for 30 and 50 mg of zein, respectively). The percentage of coumarin-6 entrapment within the nanoparticles was very high (80.0 ± 1.9% and 83.7 ± 1.8% for 30 and 50 mg of zein weight), with the highest (92.0 ± 0.9%) achieved when using 40 mg zein. Considering these findings, a 40 mg amount of zein was therefore selected for subsequent experiments, due to the smallest resultant particle size and the highest percentage of drug entrapment.

#### 3.2.7. Variation in Drug Amount Related to Zein Weight

Nanoparticles entrapping coumarin-6 were prepared by employing two distinct percentages of coumarin amount (0.1 and 0.5%) relative to the zein weight.

Notably, the nanoparticles formulated with a drug amount of 0.1% of zein weight displayed a reduced size of 201.2 ± 6.7 nm, compared with the nanoparticles prepared with 0.5% of coumarin to zein weight (215.9 ± 6.8 nm). However, their zeta potential was similar (−17.2 ± 0.8 mV and −18.4 ± 0.5 mV for 0.1% and 0.5% of the drug amount added, respectively) (Table 1). Both formulations exhibited narrow size distributions (with PDIs lower than 0.2 for both cases). However, a significant contrast emerged in terms of drug entrapment, with a mere 10.0 ± 0.2% of the drug being entrapped in the 0.1% coumarin-6 formulation, as opposed to the substantially higher drug entrapment (92.0 ± 0.9%) achieved with the 0.5% coumarin-6 formulation. In light of these observations, a coumarin amount of 0.5% relative to zein weight was selected for subsequent experiments.

The optimized formulation of zein-based hybrid lipid nanoparticles entrapping coumarin-6 was therefore prepared using the nanoprecipitation method, coupled with probe sonication. This optimal formulation encompassed key parameters, including a zein weight of 40 mg, a water–ethanol volume ratio of 2:1, and a lipid–zein weight ratio of 1:5. Additionally, the formulation incorporated a 0.5% drug content relative to the zein weight, achieved by adding 100 μL from a 2 mg/mL stock of coumarin-6.

### 3.3. Preparation of Transferrin-Bearing Zein-Based Hybrid Lipid Nanoparticles Encapsulating Docetaxel

**Figure 1:**
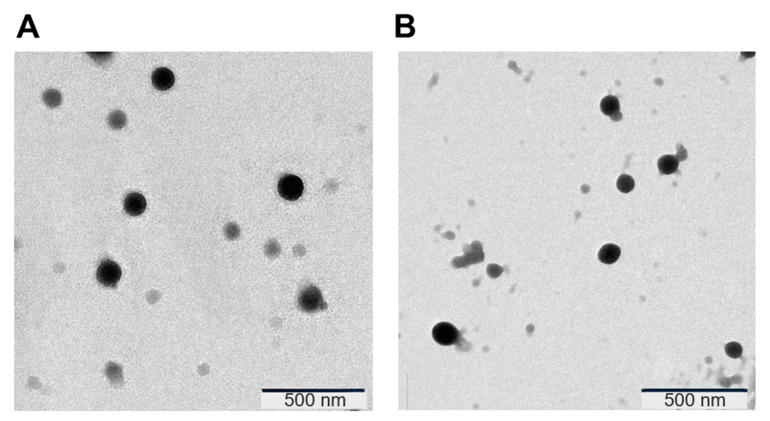
TEM pictures of (**A**) transferrin-bearing and (**B**) control zein-based hybrid lipid nanoparticles.

Transferrin-bearing zein-based hybrid lipid nanoparticles entrapping docetaxel were successfully prepared. TEM images revealed that they had a spherical shape (Figure 1).

The entrapment efficiency of docetaxel within the nanoparticles was relatively high, respectively, 59.6 ± 2.1% and 67.7 ± 1.8% of docetaxel for Tf-bearing and control nanoparticles (equivalent to 119.2 ± 1.7 mg and 135.4 ± 1.2 µg of docetaxel, respectively).

Tf was conjugated to the nanoparticles at the substantial level of 66.9 ± 0.8% of the initial Tf (equivalent to 6.7 ± 0.5 mg of Tf per mL of nanoparticles).

The conjugation of Tf to the surface of the nanoparticles led to an increase in the hydrodynamic size of the targeted nanoparticles (344.2 ± 15.9 nm, with a PDI of 0.24 ± 0.03). In contrast, the size of control nanoparticles was notably smaller, measuring 182.0 ± 2.5 nm with a PDI of 0.12 ± 0.02. It also marginally decreased the net surface charge of Tf-bearing nanoparticles (−28.9 ± 0.5 mV), relative to the control formulation (−23.3 ± 1.4 mV).

### 3.4. Stability of the Formulations

**Figure 2:**
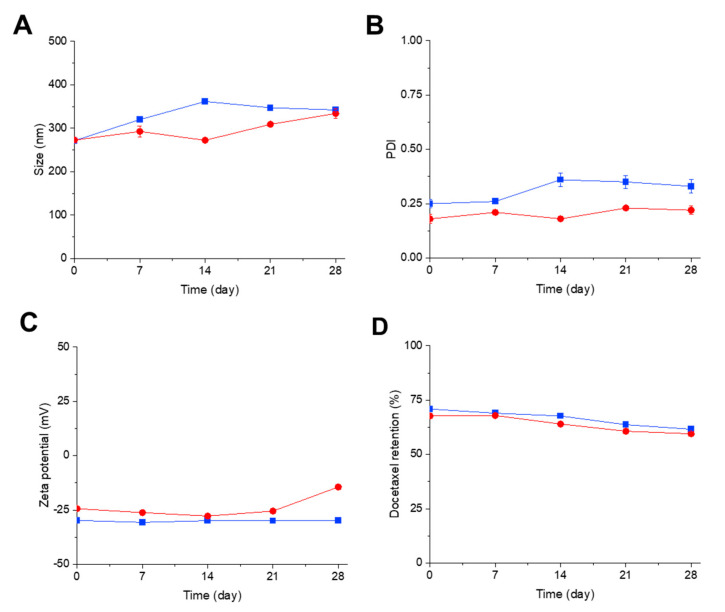
Size (**A**), polydispersity index (**B**), zeta potential (**C**), and drug retention (**D**) of Tf-bearing (■, blue) and control (●, red) zein-based hybrid lipid nanoparticles entrapping docetaxel after storage at 4 °C for 4 weeks (*n* = 4) (error bars smaller than symbols when not visible).

Transferrin-bearing and control zein-based hybrid lipid nanoparticles entrapping docetaxel were found to be stable when stored at 4 °C for a duration of 4 weeks. They displayed a slight increase in size and polydispersity index over the course of 28 days (from 271.6 ± 6.1 nm (PDI: 0.25 ± 0.02) on Day 0 to 342.2 ± 1.4 nm (PDI: 0.33 ± 0.03) on Day 28 for Tf-bearing formulations; from 272.8 ± 5.7 nm (PDI: 0.18 ± 0.02) on Day 0 to 334.1 ± 10.8 nm (PDI: 0.22 ± 0.02) on Day 28 for control formulations) (Figure 2A,B).

The zeta potential of the targeted formulation remained stable throughout the entire experiment (−29.8 ± 0.3 mV on Day 0 and −29.9 ± 0.6 mV on Day 28), in contrast to control nanoparticles, which displayed an increase in zeta potential (from −24.4 ± 0.0 mV on Day 0 to −14.4 ± 0.6 mV on Day 28) (Figure 2C).

In terms of drug leakage, the percentage of drug retention, although gradually decreasing, remained notably high over the 4-week period (reducing from 70.9 ± 1.0% on Day 0 to 61.6 ± 0.9% on Day 28 for targeted nanoparticles, and from 67.7 ± 0.8 on Day 0 to 59.5 ± 0.4% on Day 28 for control nanoparticles) (Figure 2D).

### 3.5. Drug Release

**Figure 3:**
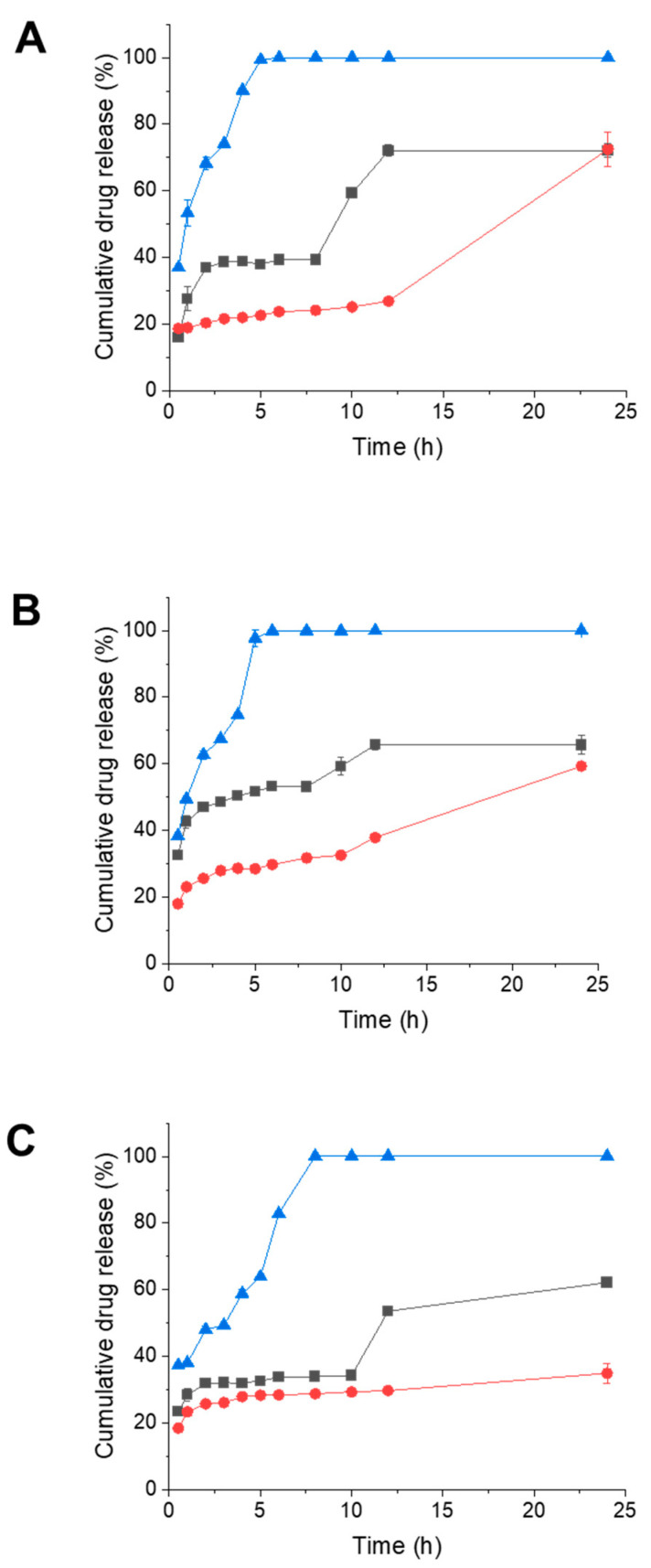
Drug release profile of docetaxel entrapped in Tf-bearing (■, black), control zein-based hybrid lipid nanoparticles (●, red), or in solution (▲, blue) in phosphate buffers at pH 5.5 (**A**), 6.5 (**B**), and 7.4 (**C**) over 24 h (*n* = 3) (error bars smaller than symbols when not visible).

The Tf-bearing and control zein-based hybrid lipid nanoparticles consistently demonstrated sustained drug release profiles across pH levels of 5.5, 6.5, and 7.4, following an initial burst release phase. Notably, the drug released from the solution exhibited rapid diffusion through the dialysis membrane, resulting in complete release within 8 h (Figure 3).

At pH 5.5, transferrin-bearing nanoparticles exhibited a gradual initial release of docetaxel, with 40% of the maximum release occurring within the first 8 h (Figure 3A). Subsequently, the drug release then increased, reaching its highest percentage (72%) at 12 h. In comparison, control nanoparticles displayed a slower drug release within the first 12 h (peaking at 26.9% of docetaxel released), followed by a sharp surge in release to match the levels of the Tf-bearing formulation (72% of the drug released at 24 h). Conversely, docetaxel in solution was fully released from the dialysis tubing within 5 h (Figure 3A).

At pH 6.5, transferrin-bearing nanoparticles led to a faster drug release than at pH 5.5, with 53% of the drug released within the initial 8 h and reaching 65% at 12 h (Figure 3B). In contrast, control nanoparticles exhibited a slower drug release profile compared to transferrin-bearing nanoparticles (with only 59.3% of the drug released at 24 h), while docetaxel in solution reached complete release within 6 h. The cumulative drug release percentages for both transferrin-bearing and control nanoparticles were lower than those observed at pH 5.5 (Figure 3B).

At pH 7.4, transferrin-bearing nanoparticles resulted in an even more restrained drug release compared to pH 5.5 and 6.5. Over the initial 10 h, the drug exhibited a gradual release (34% released) to reach its highest percentage of 62.1% at 24 h (Figure 3C). Control nanoparticles displayed a similarly limited drug release, with only 34.9% of the drug released within 24 h. In contrast, docetaxel in solution attained complete release in 7 h.

Overall, the highest cumulative drug release percentages were observed at pH 5.5 for both transferrin-bearing and control nanoparticles (Figure 3).

### 3.6. In Vitro Studies

#### 3.6.1. Cellular Uptake

**Figure 4:**
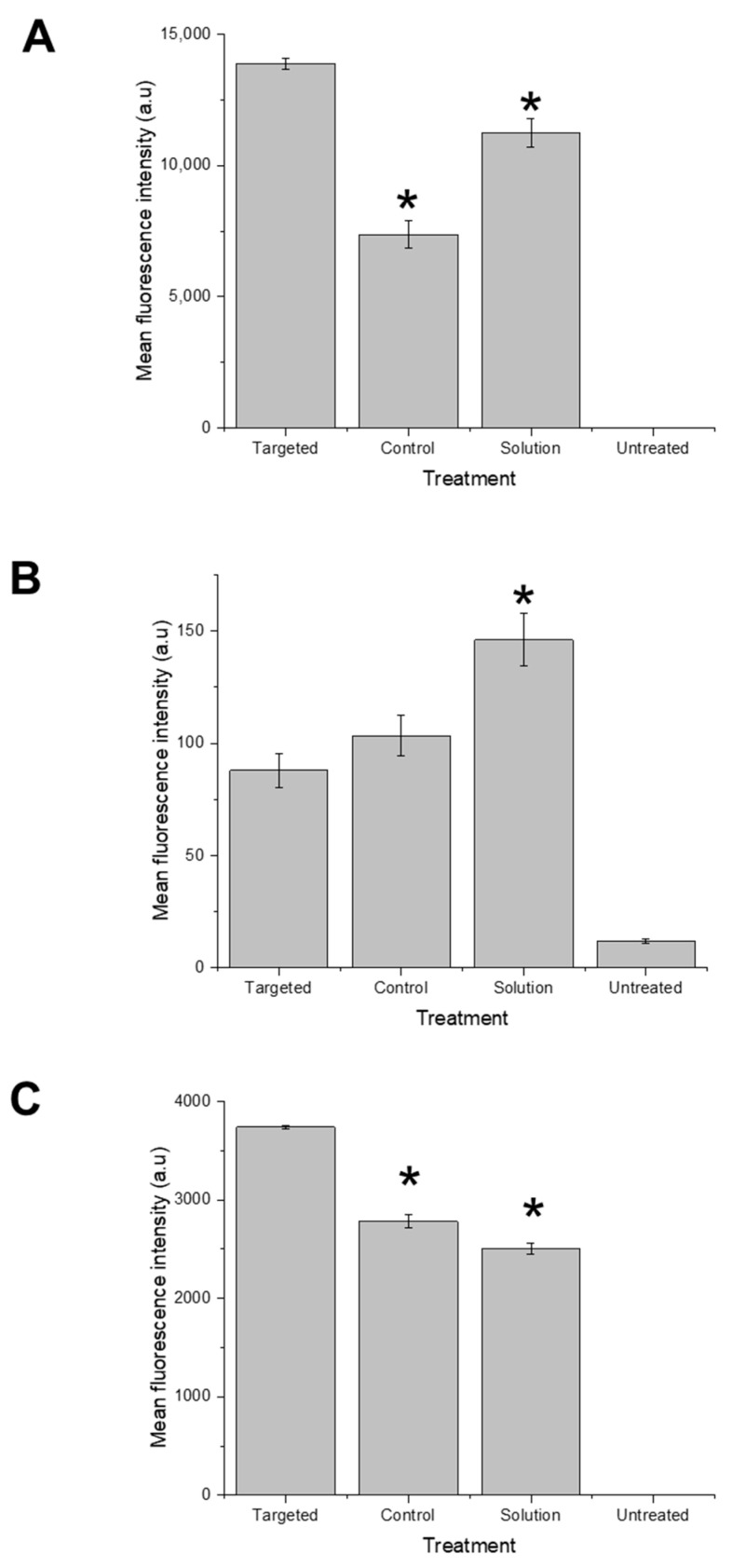
Quantification of the cellular uptake of fluorescein-labelled docetaxel entrapped in Tf-bearing and control zein-based hybrid lipid nanoparticles or as solution in PC3-Luc (**A**), DU145 (**B**), and LNCaP (**C**) cells (a.u.: arbitrary units) (*: *p* < 0.05 vs. Tf-bearing zein-based hybrid lipid nanoparticles). Results represent mean ± SEM of 3 independent experiments.

The entrapment of docetaxel in Tf-bearing zein-based hybrid lipid nanoparticles significantly increased the drug uptake by both PC-3 and LNCaP cancer cell lines compared to control nanoparticles and drug solution (Figure 4).

In PC3-Luc cells, the drug uptake was higher than that of the control nanoparticles and the drug solution by 1.8-fold and 1.2-fold, respectively (13,880 ± 221 a.u for Tf-bearing nanoparticles, 7366 ± 520 a.u for control nanoparticles, and 11,239 ± 532 a.u. for the drug solution) (Figure 4A).

In contrast, within DU145 cells, the treatment with docetaxel solution yielded the highest cellular uptake, exhibiting a mean fluorescence intensity of 146 ± 11.67 a.u., in contrast to 88 ± 7.57 a.u. and 103.3 ± 8.9 a.u. for docetaxel entrapped in targeted and non-targeted nanoparticles, respectively (Figure 4B).

In the case of LNCaP cells, the conjugation of transferrin to the nanoparticles notably enhanced the cellular uptake of fluorescein-labelled docetaxel, leading to a 1.3-fold increase in mean fluorescence intensity (3740 ± 16 a.u.) compared to control nanoparticles (with a mean fluorescence intensity of 2783 ± 64 a.u.) (Figure 4C). Conversely, the uptake of docetaxel solution was comparatively the lowest, with a mean fluorescence intensity of 2504 ± 56 a.u.

These findings correlated well with the observations made through confocal microscopy.

**Figure 5:**
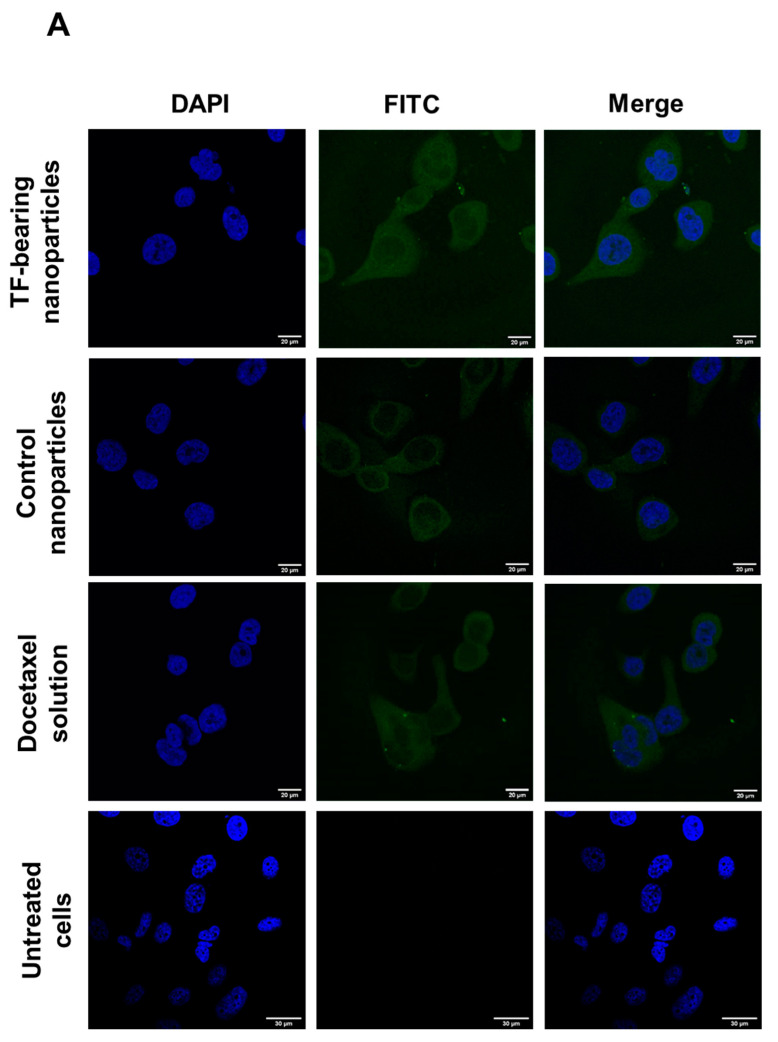

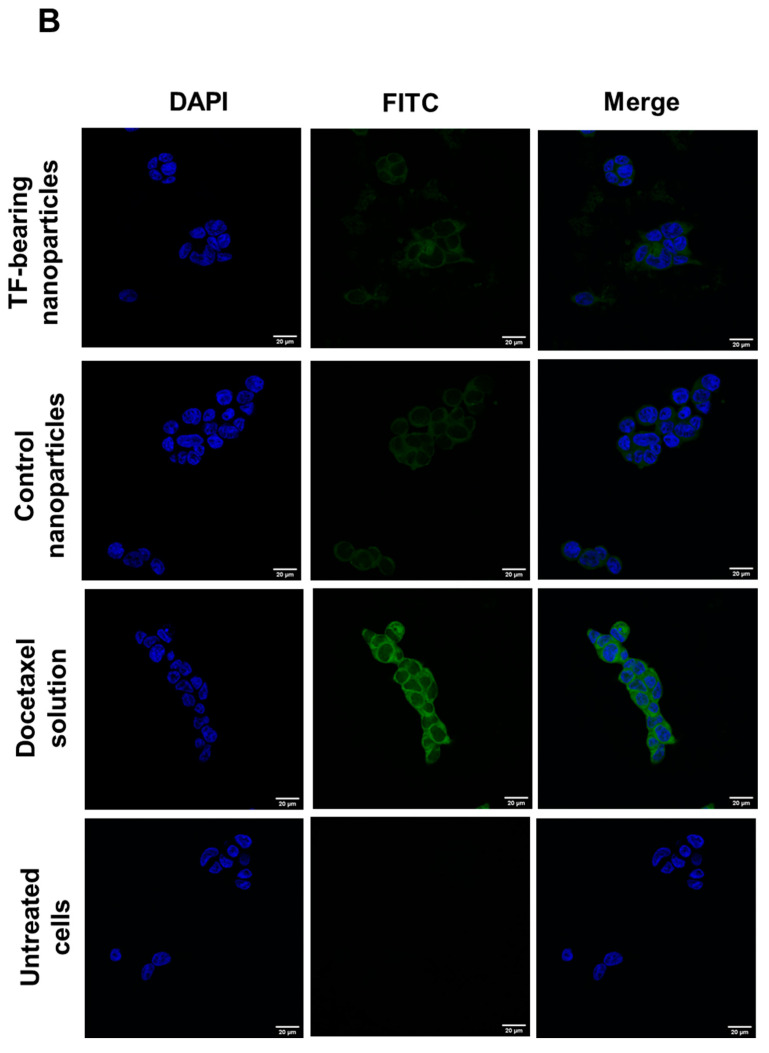

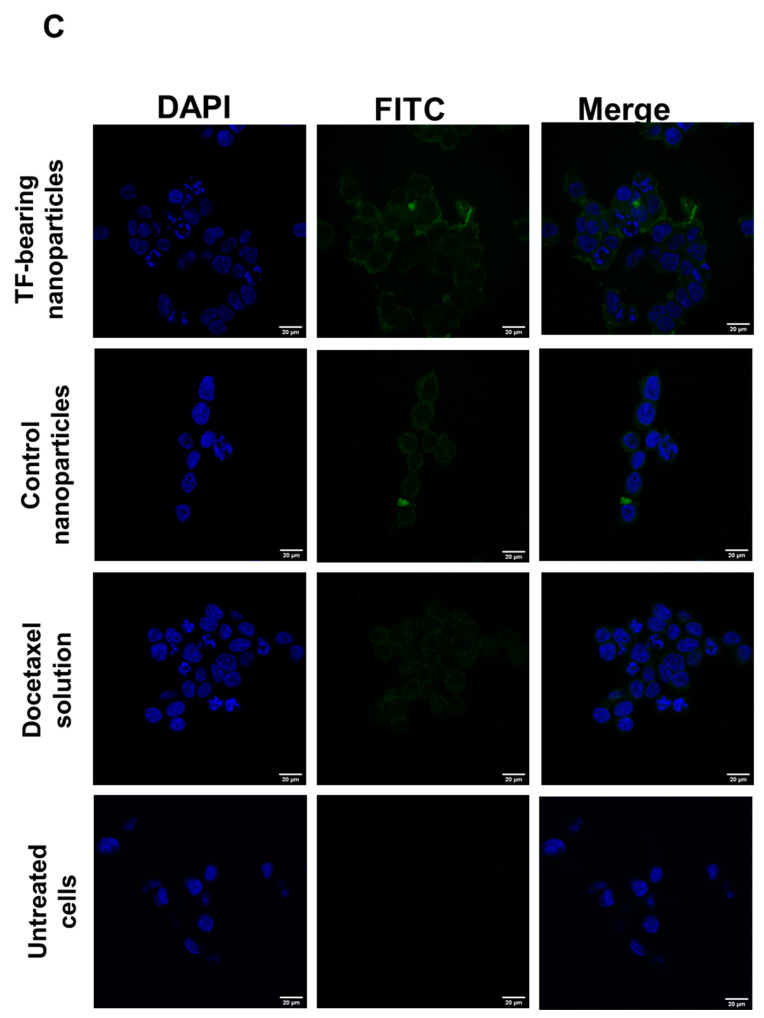
Confocal microscopy images of the cellular uptake of fluorescein-labelled docetaxel (at a concentration of 12 μg/well) entrapped in transferrin-bearing and control zein-based hybrid lipid nanoparticles as well as in solution, after incubation for 15 h with PC3-Luc (**A**), DU145 (**B**), and LNCaP cells (**C**). Blue: nuclei stained with DAPI (excitation: 405 nm laser line, bandwidth: 400–480 nm), green: fluorescein-labelled docetaxel (excitation: 494 nm laser line, bandwidth: 510–530 nm) (bar size: 20 μm).

In PC-3-Luc cells, the treatment with transferrin-bearing zein-based hybrid lipid nanoparticles yielded a notably higher cellular uptake of docetaxel, contrasting against the uptake seen with control nanoparticles (Figure 5A). The fluorescence intensity associated with cellular uptake following drug solution treatment also appeared substantial.

For DU145 cells, the most pronounced fluorescence intensity was observed in cells treated with docetaxel in solution, followed by cells treated with control zein-based hybrid lipid nanoparticles, with transferrin-bearing nanoparticles entrapping fluorescein-labelled docetaxel displaying comparatively lower fluorescence intensity (Figure 5B).

Within LNCaP cells, the peak fluorescence intensity was observed in cells treated with docetaxel entrapped within the targeted delivery system. This was followed by cells treated with docetaxel entrapped within the control delivery system (Figure 5C).

Throughout these treatments, fluorescein-labelled docetaxel showcased distribution within the cell cytoplasm, with no observable co-localization within the nucleus.

#### 3.6.2. Mechanisms of Cellular Uptake

**Figure 6:**
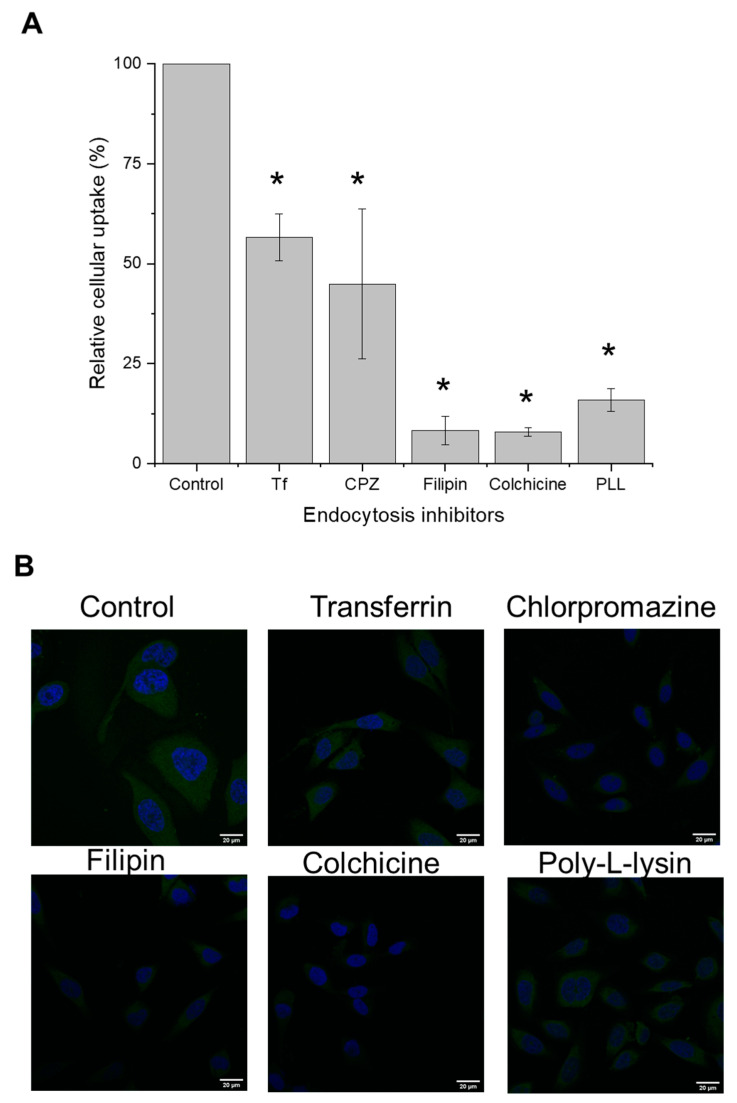
Relative cellular uptake of fluorescein-labelled docetaxel entrapped in Tf-bearing zein-based hybrid lipid nanoparticles by PC3-Luc cells in the presence of endocytosis inhibitors, using flow cytometry (**A**) and confocal microscopy (**B**) (*: *p* < 0.05 vs. no inhibitor). Results represent mean ± SEM of 3 repeats.

The uptake of docetaxel entrapped within transferrin-bearing zein-based hybrid lipid nanoparticles was significantly decreased by both filipin and colchicine, resulting in cellular uptake inhibitions of 92.0% and 92.4%, respectively. These outcomes suggest that the primary mechanism of internalization involves caveolae-mediated endocytosis and macropinocytosis (Figure 6).

Pre-treatment of the cells with PLL, which acts as an uptake inhibitor for cationic delivery systems, resulted in a notable 84.5% reduction in cellular uptake, thus demonstrating the influence of nanoparticle charges on the internalization of docetaxel entrapped within transferrin-bearing nanoparticles.

In addition, the nanoparticles uptake was partially inhibited by free transferrin and chlorpromazine (with inhibitions of 45 and 59%, respectively), suggesting that this delivery system was also internalized through transferrin receptors and clathrin-mediated endocytosis (Figure 6A). Confocal microscopy imaging of cellular uptake mechanisms yielded similar trends (Figure 6B).

#### 3.6.3. Anti-Proliferative Efficacy

**Figure 7:**
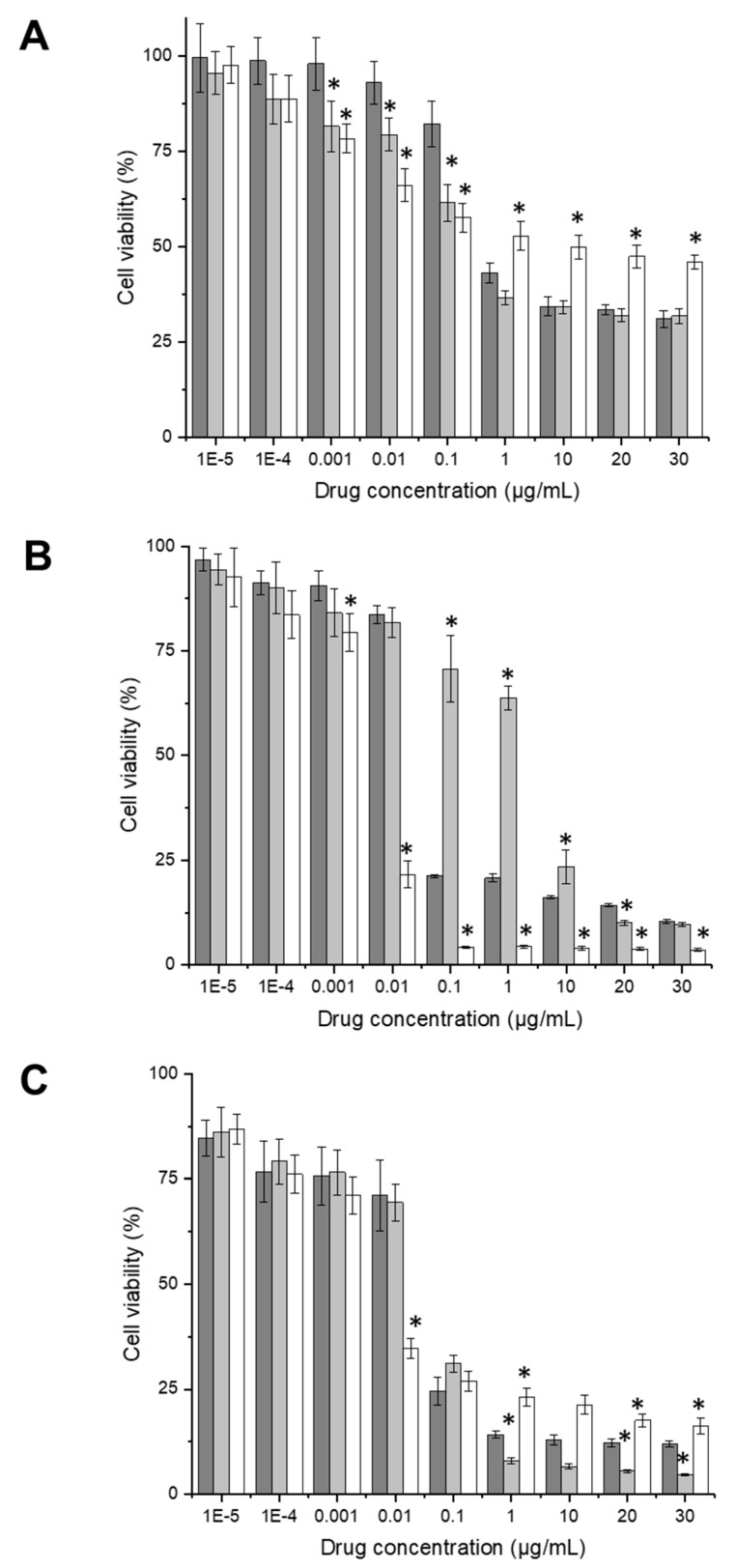
Anti-proliferative efficacy of docetaxel entrapped in Tf-bearing zein-based hybrid lipid nanoparticles (dark grey), control lipid nanoparticles (grey), or in solution (white), on PC3-Luc (**A**), DU145 (**B**), and LNCaP (**C**) cells (*: *p* < 0.05 vs. the drug concentration leading to the highest cell viability) (*n* = 15).

In PC3-Luc cells, treatment of the cells with docetaxel entrapped within transferrin-bearing zein-based hybrid lipid nanoparticles resulted in a significant increase in anti-proliferative efficacy compared to docetaxel solution, when using docetaxel concentrations exceeding 1 µg/mL (Figure 7A). Both Tf-bearing and control formulations showcased similar anti-proliferative efficacy. At a drug concentration of 30 µg/mL, the percentages of cell viability were 31.0 ± 2.1% and 31.8 ± 1.9% for Tf-bearing and control formulations, respectively, whereas treatment with docetaxel solution yielded a higher cell viability of 45.9 ± 1.9%.

In DU145 cells, the conjugation of Tf to the nanoparticles led to a significantly enhanced antiproliferative efficacy at drug concentrations of 0.1, 1, and 10 µg/mL, although the most potent formulation at these concentrations remained docetaxel solution (Figure 7B). At the highest drug concentration tested (30 µg/mL), the cell viability was 10.3 ± 0.4% and 9.6 ± 0.3% following treatment with Tf-bearing and control nanoparticles, which was about 3-fold higher than that resulting from docetaxel solution treatment (3.6 ± 0.2%).

In LNCaP cells, docetaxel nanoparticle formulations at drug concentrations of 1 µg/mL and above demonstrated increased anti-proliferative efficacy when compared to the drug solution, with the highest efficacy observed following treatment with the control nanoparticles (Figure 7C). At the highest tested docetaxel concentration (30 µg/mL), the percentages of cell viability were 12.0 ± 0.7%, 4.6 ± 0.3%, and 16.3 ± 1.9% for cells treated with docetaxel entrapped in Tf-bearing, control zein-based hybrid lipid nanoparticles, and docetaxel in solution, respectively.

The anti-proliferative efficacy of docetaxel entrapped within transferrin-bearing zein-based hybrid lipid nanoparticles exhibited a ranking order of higher efficacy in DU145 cells, followed by LNCaP cells, and then PC3 cells, with respective reductions in cell viability of 89.7%, 88%, and 70%.

Docetaxel entrapped within Tf-bearing lipid nanoparticles exhibited notably higher anti-proliferative efficacy in comparison to control nanoparticles when evaluated on DU145 and LNCaP cells (IC_50_: 0.02 ± 0.01 µg/mL vs. 1.96 ± 0.54 µg/mL for Tf-bearing and control formulations in DU145 cells; 0.02 ± 0.00 µg/mL vs. 0.05 ± 0.01 µg/mL for Tf-bearing and control formulations in LNCaP cells). However, despite these enhancements, docetaxel solution continued to demonstrate the utmost anti-proliferative efficacy across all three cell lines, displaying its most minimal IC_50_ of 0.0006 ± 8 × 10^−4^ µg/mL in PC3 cells (Table 2).

### 3.7. Evaluation of the Potential Use of Tf-Bearing Zein-Based Hybrid Lipid Nanoparticles for Gene Therapy

#### 3.7.1. Evaluation of DNA Condensation

**Figure 8:**
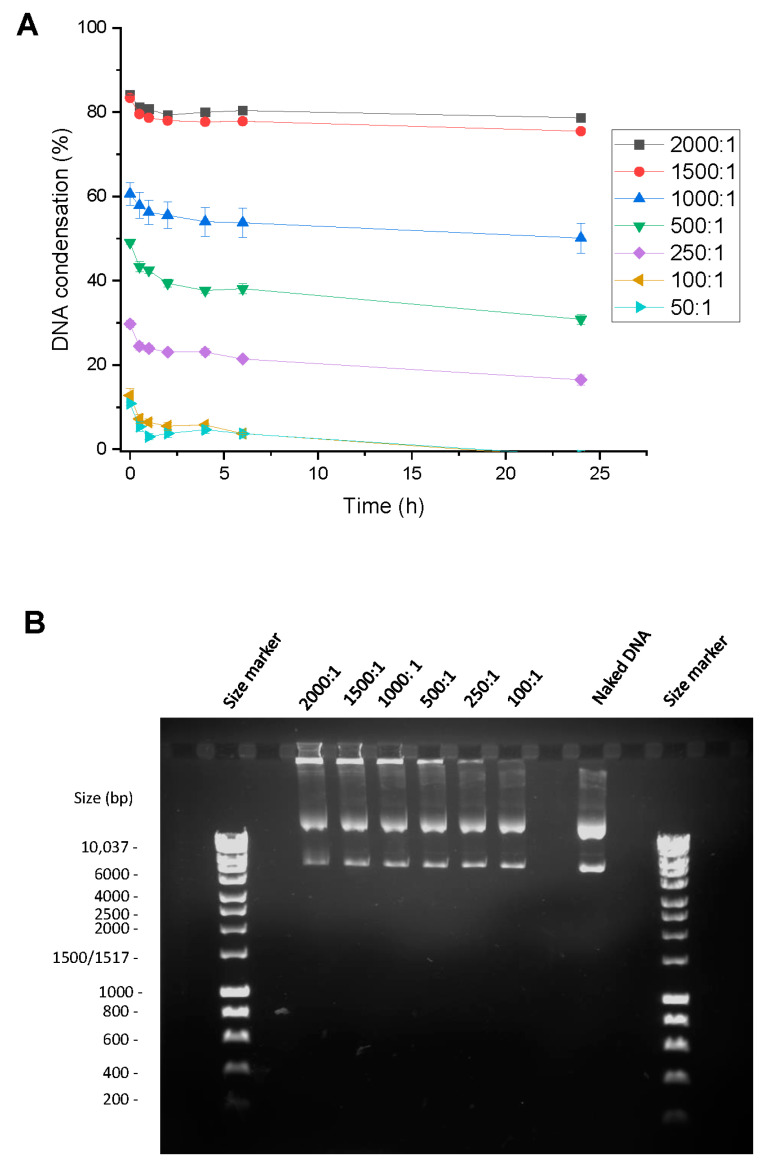
DNA condensation to Tf-bearing zein-based hybrid lipid nanoparticles at various polymer–DNA ratios, using PicoGreen^®^ assay (**A**) (*n* = 4) and gel retardation assay (**B**) (error bars smaller than symbols when not visible).

Tf-bearing zein-based hybrid lipid nanoparticles effectively condensed over 80% of DNA at polymer–DNA weight ratios of 1500:1 and 2000:1, showcasing condensation capabilities of 83% and 84%, respectively (Figure 8A). This DNA condensation occurred instantly and exhibited stability for a minimum of 24 h. As the polymer–DNA weight ratios decreased, the level of DNA condensation also decreased. At lower polymer–DNA weight ratios of 500:1 and 1000:1, it dropped to 49% and 60%, respectively. At even lower ratios ranging from 250:1 to 50:1, DNA complexation was notably limited, ranging from 29% to 10%.

A gel retardation assay confirmed the complete and partial condensation of DNA by Tf-bearing zein-based hybrid lipid nanoparticles (Figure 8B). At polymer–DNA weight ratios of 1500:1 and 2000:1, DNA was predominantly condensed by Tf-bearing zein-based hybrid lipid nanoparticles, effectively inhibiting the intercalation of ethidium bromide with DNA. Consequently, minimal DNA was visually discernible at these ratios. Conversely, at polymer–DNA ratios of 250:1 and 100:1, DNA underwent partial condensation by transferrin-bearing nanoparticles. This allowed for ethidium bromide to intercalate with DNA, resulting in a prominent band indicative of free DNA at these two ratios.

#### 3.7.2. Measurement of the Size and Zeta Potential of the Complexes

**Figure 9:**
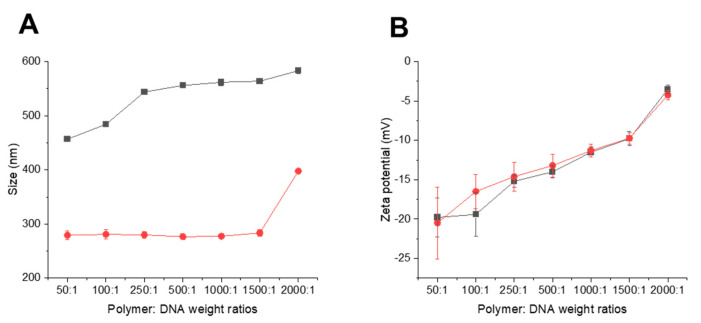
Size (**A**) and zeta potential (**B**) of transferrin-bearing zein-based hybrid lipid nanoparticles (black, ■) and control zein-based hybrid lipid nanoparticles (red, ●) complexed with DNA at various polymer–DNA weight ratios. Results are expressed as mean ± SEM (*n* = 4) (error bars smaller than symbols when not visible).

Transferrin-bearing zein-based hybrid nanoparticles complexed with DNA consistently displayed an average size of less than 600 nm across all polymer–DNA weight ratios investigated (Figure 9A). Their size increased with increasing weight ratios used (Figure 9A), spanning from 456.7 ± 3.0 nm at a polymer–DNA weight ratio of 50:1 to 583.1 ± 48.8 nm at a ratio of 2000:1.

Conversely, the size of control zein-based hybrid nanoparticles complexed with DNA remained stable within the range from 50:1 to 1500:1 ratios, measuring between 279.2 ± 8.1 nm and 283.5 ± 6.6 nm. It increased at the higher polymer–DNA ratio of 2000:1, reaching 397.7 ± 0.5 nm (Figure 9A).

Both transferrin-bearing and control zein-based hybrid nanoparticles complexed with DNA were bearing a negative surface charge, which increased with increasing polymer–DNA ratios (from −19.8 ± 2.5 mV DNA at a weight ratio of 50:1 to −3.5 ± 0.5 mV at a weight ratio of 2000:1 for the transferrin-bearing complex; from −20.5 ± 4.5 mV to −4.2 ± 0.5 mV at 50:1 and 2000:1 ratios for the control complex (Figure 9B).

#### 3.7.3. Gene Expression

**Figure 10:**
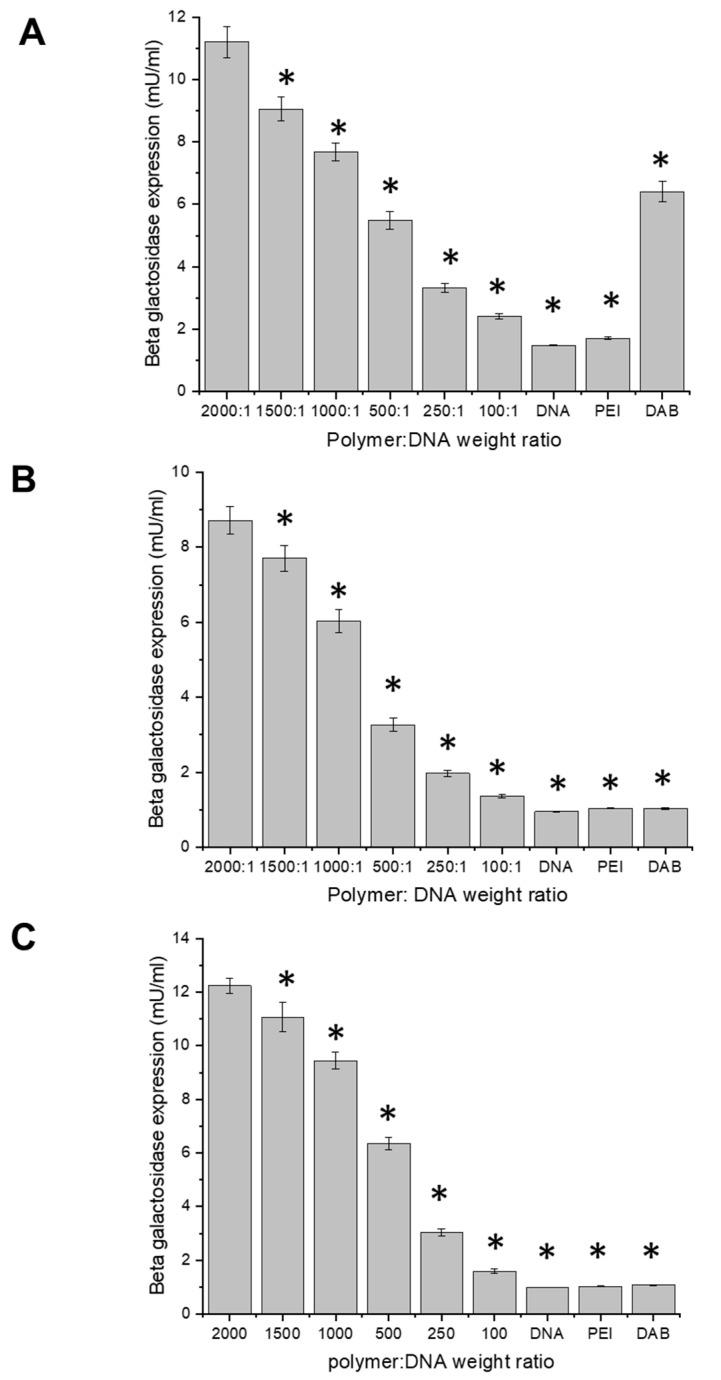
Gene expression efficacy of transferrin-bearing zein-based hybrid lipid nanoparticles in PC3-Luc (**A**), DU145 (**B**), and LNCaP cells (**C**). Results are expressed as mean ± SEM (*n* = 15), using DAB–DNA and PEI–DNA as positive controls (*: *p* < 0.05 vs. the polymer–DNA ratio leading to the highest transfection).

Transferrin-bearing zein-based hybrid lipid nanoparticles complexed to a plasmid DNA encoding β-galactosidase exhibited increased gene expression at optimal polymer–DNA ratios across the three tested cell lines, in comparison to DAB–DNA and PEI–DNA complexes (Figure 10). The highest level of transfection was achieved at polymer–DNA weight ratios of 2000:1 for all three cell lines (11.19 ± 0.48 mU/mL in PC3-Luc cells, 8.71 ± 0.37 mU/mL in DU145 cells, and 12.23 ± 0.29 mU/mL in LNCaP cells). This result marked a substantial increase of 1.7-fold, 6.7-fold, and 11.4-fold when compared to DAB–DNA in PC3-Luc, DU145, and LNCaP cells; and 6.5-fold, 8.3-fold, and 11.9-fold in comparison to PEI–DNA in the corresponding cell lines. Notably, the highest transfection efficacy was observed in LNCaP cells, followed by PC-3-Luc cells.

In PC3-luc cells, the three highest ratios of transferrin-bearing zein-based hybrid lipid nanoparticles complexed to a plasmid DNA led to an increased gene expression relative to DAB–DNA. Furthermore, all the tested ratios of this formulation resulted in increased transfection compared to PEI–DNA (Figure 10A). Similarly, in DU145 and LNCaP cells, the transferrin-bearing complex at all ratios led to an increased gene expression compared to PEI–DNA and DAB–DNA (Figure 10B,C).

## 4. Discussion

The potential application of nanomedicines in cancer therapy is currently hindered by the limitations in existing delivery systems. These systems often fail to achieve precise targeting, leading to adverse effects on healthy tissues [34]. Additionally, they struggle to encapsulate hydrophobic anti-cancer drugs, which constitute over 40% of emerging pharmaceuticals [35], and effectively transport therapeutic nucleic acids. Furthermore, the complexity of their manufacturing methods imposes further restrictions on their practical application [36].

To address these challenges, we hypothesize that hybrid lipid nanoparticles made of biocompatible and biodegradable zein and conjugated to transferrin (whose receptors are overexpressed on cancer cells), able to entrap the hydrophobic anti-cancer drug docetaxel and carry plasmid DNA, would enhance the delivery of the therapeutic payloads to prostate cancer cells and increase their anti-proliferative efficacy and gene expression levels.

In this study, coumarin-6 was entrapped within zein-based hybrid lipid nanoparticles. Zein, a hydrophobic and biodegradable protein extracted from corn, was used to form the polymeric core of these hybrid nanoparticles. The core effectively entrapped coumarin-6, while a lipid monolayer composed of DSPE-PEG2K-MAL surrounded it. This lipid layer served multiple functions, including conferring a stealth effect and facilitating surface modification [37,38,39].

The lipid nanoparticles were successfully prepared using nanoprecipitation, where the zein dissolved in ethanol was mixed with the lipid dispersed in water to allow for self-assembling. Mixing these two solutions caused a decrease in the concentration of the organic solvent (ethanol) within the dissolved zein solution. This reduction triggered processes such as desolvation, phase separation, and ultimately, the formation of nanoparticles. In addition, the one-step nanoprecipitation method exhibited distinct advantages over alternative techniques for nanoparticle generation, such as the two-step nanoprecipitation method. This one-step approach consumed less time and energy. Due to its efficiency, it has gained widespread popularity for encapsulating various drugs [40,41].

Among the various formulations investigated, zein-based hybrid lipid nanoparticles entrapping coumarin-6 (used as a fluorescent lipophilic drug model) prepared using the nanoprecipitation method, coupled with probe sonication, formulated with a zein weight of 40 mg, a water–ethanol volume ratio of 2:1, and a lipid–zein weight ratio of 1:5, and incorporating a 0.5% drug content relative to the zein weight, yielded optimal results. They were found to exhibit a compact particle size (215.9 ± 6.8 nm), with a low PDI (0.16 ± 0.04). Additionally, they displayed a negative zeta potential (−18.4 ± 0.5 mV), while maintaining a high entrapment of coumarin-6 (92.0 ± 0.9%). These hybrid lipid nanoparticles had a size smaller than the critical threshold for extravasation (which has been determined to be around 400 nm for most tumors [33]) and therefore have the required properties to access the cancer cells and deliver the entrapped drug. Moreover, the negative charge they carried further contributed to their efficacy. This negative charge would diminish the likelihood of electrostatic interactions with negatively charged cell membranes, therefore reducing any non-specific uptake by non-cancerous cells [42].

The optimal formulation of zein-based hybrid lipid nanoparticles was obtained when using a low water to organic solvent volume ratio of 2:1, which resulted in a small particle size while concurrently maintaining a high entrapment of coumarin-6 (exceeding 90%). Increasing the volume ratio of water to organic solvent caused a significant decrease in the entrapment efficiency of coumarin-6, as the drug exhibits poor solubility similarly to our findings when optimizing transferrin-targeted PEGylated nanoparticles entrapping the hydrophobic plumbagin [43].

Low lipid to zein weight ratios were needed to achieve an optimal formulation of zein-based hybrid lipid nanoparticles to ensure that the surface of the zein core was enveloped by the lipids [44]. When employing high lipid to zein weight ratios, the surplus of DSPE-PEG2K-MAL lipids would have the potential to spontaneously form liposomes. This outcome would lead to a noticeable increase in the overall hydrodynamic size of the nanoparticles and a subsequent reduction in their zeta potential value.

The specific arrangements of PEG chains at varying grafting densities could also influence the size of the delivery system [45,46]. When present at a low density (10–30 mol%), the PEG chains adopted a mushroom-like conformation which had a limited effect on the size and zeta potential of the particles. In contrast, higher grafting densities prompted the PEG chains to stretch and form a brush-like structure, leading to an increase in the size and zeta potential of the particles.

Subsequently, the zein-based hybrid lipid nanoparticles were then conjugated with transferrin using the thiol–maleimide ‘click’ reaction, a commonly used thiol-based bioconjugation technique for conjugating antibodies, proteins, or peptides to delivery systems. Its popularity stems from its compatibility with aqueous environments, swift reaction kinetics (without necessitating heat or catalysts), and high selectivity [47]. Traut’s reagent (2-iminothiolane) was used as a cross-linking agent to produce a thiolated form of Tf by thiolation of the amino group present within the transferrin, which could subsequently interact with the thiol-reactive maleimide group. As a result of this procedure, the conjugation process was successful, with a yield of 66.9 ± 0.8% of the initial Tf being effectively conjugated. While no existing literature detailing the formulation of a transferrin-bearing zein-based delivery system was found for comparative purposes, our results aligned with prior findings from our research group. This consistency was observed across various delivery systems previously explored by our group in the context of Tf conjugation [9].

The conjugation of transferrin to the surface of nanoparticles led to a noticeable increase in the mean diameter of the nanoparticles when compared with control nanoparticles, due to the substantial molecular weight of transferrin (ranging from 70,000 to 80,000 Daltons) [48]. Nonetheless, it is essential to note that both the transferrin-bearing and control nanoparticles maintained sizes that remained beneath the threshold necessary for extravasation through the tumor tissue vessels (approximately 400 nm for solid tumors).

Transferrin-bearing hybrid lipid nanoparticles exhibited negative surface charges, attributed to the presence of transferrin itself (with a zeta potential of −28.9 ± 0.5 mV). These negative charges, in conjunction with the PEGylation of the formulation, would effectively prevent the opsonization of the nanoparticles by serum proteins, therefore avoiding a rapid clearance by the mononuclear phagocytic system and extending their circulation time within the bloodstream [49,50]. Furthermore, the transferrin-bearing hybrid lipid nanoparticles could be classified as moderately stable colloidal systems, as their zeta potential value falls within the range from −20 to −30 mV [51].

The entrapment efficiency of docetaxel within the nanoparticles was relatively high, respectively, at 59.6 ± 2.1% and 67.7 ± 1.8% of docetaxel for Tf-bearing and control nanoparticles (equivalent to 119.2 ± 1.7 mg and 135.4 ± 1.2 µg of docetaxel, respectively). This was slightly higher than that reported when entrapping docetaxel within zein nanoparticles coated by a green tea polyphenols/iron coordination complex (45.0 ± 3.1%) [52]. However, it remained below the levels observed when employing glucose-bearing zein nanoparticles (85.23 ± 3.15%) [53], folate-conjugated zein/soy lecithin/carboxymethyl chitosan core−shell nanoparticles (86.75 ± 1.61%) [54], or chondroitin sulfate-hybridized zein nanoparticles (64.2 ± 1.9%) [55]. We were unable to find any existing publications detailing the entrapment efficiency of docetaxel within hybrid lipid nanoparticles for the purpose of comparison with our findings. Moreover, when stored at 4 °C over a span of 4 weeks, the transferrin-bearing hybrid lipid nanoparticles demonstrated minimal alterations in size and drug leakage. This stability over time and under these storage conditions is an encouraging indicator of the robustness of the formulation.

Transferrin-bearing hybrid lipid nanoparticles displayed a sustained release profile of docetaxel, likely attributed to the diffusion of the drug entrapped within the zein core through water-filled pores. This diffusion-based mechanism of release is a widely recognized phenomenon in polymer-based nanoparticles [56].

It was initially anticipated that the Tf-bearing nanoparticles would exhibit a comparatively slower drug release when compared with the control formulation. This expectation arose from the hypothesis that the conjugated transferrin might hinder the diffusion of the drug out of the zein core. However, that was not the case in this study. The observed release profile was consistent with findings from other studies involving chondroitin sulfate-hybridized zein nanoparticles, folate-conjugated zein/soy lecithin/carboxymethyl chitosan core−shell nanoparticles, and glucose-bearing zein nanoparticles. In all these cases, approximately 60% of the drug was released within a 24 h timeframe [53,54,55]. This similarity suggests a common release pattern among these formulations.

The cellular uptake studies revealed a significant increase in drug uptake upon the conjugation of transferrin to zein-based hybrid lipid nanoparticles, particularly in comparison with the control formulation and the drug solution on PC-3 and LNCaP cancer cell lines. These findings were in accordance with earlier research conducted by Guo and colleagues. In their study, the use of transferrin as a targeting ligand on lipid-coated PLGA nanoparticles entrapping doxorubicin led to a substantial 2.8-fold improvement in cellular uptake of doxorubicin compared to non-targeted nanoparticles in A549 cells [57]. Similar findings were also reported by Zheng and colleagues, who demonstrated that transferrin-conjugated lipid-coated PLGA nanoparticles entrapping calcein exhibited more effective uptake by SKBR-3 breast cancer cells than their non-targeted counterparts [58]. Furthermore, the presence of transferrin has been shown to impede the efflux of delivery systems from within cells, resulting in increased retention of drugs within the cells [59]. The cellular uptake of docetaxel within the targeted nanoparticles displayed the lowest rates in DU145 cells compared with the two other cell lines. This variation was attributed to the lower expression of transferrin receptors on DU145 cells, as documented by Deng and colleagues [60].

The uptake of docetaxel entrapped within transferrin-bearing zein-based hybrid lipid nanoparticles was predominantly hindered by both filipin and colchicine. Filipin, known as a pinocytosis inhibitor, blocks the caveolae-mediated process, which is a form of clathrin-independent endocytosis [61]. Colchicine has previously been shown to inhibit macropinocytosis, a non-specific process used to internalize particles and fluids [62]. These findings collectively propose that the principal mode of internalization for transferrin-bearing zein-based hybrid lipid nanoparticles encompasses two pathways: caveolae-mediated endocytosis, which is essential for Tf receptor-mediated endocytosis, and macropinocytosis.

The entrapment of docetaxel within transferrin-bearing zein-based hybrid lipid nanoparticles led to a significant increase in anti-proliferative efficacy when compared to docetaxel solution, at high docetaxel concentrations exceeding 1 µg/mL, in PC-3 cells. This could be attributed to the enhanced cellular uptake facilitated by the targeted formulation. However, in DU145 cells, the most potent formulation at high drug concentrations remained the docetaxel solution, aligning with the higher cellular uptake observed for this formulation. Intriguingly, the highest anti-proliferative efficacy emerged from the treatment with control nanoparticles in LNCaP cells. Prior research has already highlighted the capability of various zein-based delivery systems to improve the anti-proliferative efficacy of docetaxel. For example, the IC_50_ values of docetaxel solution and chondroitin sulfate-hybridized zein nanoparticles were reported as 170.7 ± 7.5 and 61.2 ± 10.4 ng/mL after 72 h of incubation in PC-3 cells. This can be partly attributed to the enhanced cellular uptake efficiency of the zein nanoparticles [55]. Similarly, in another study, the anti-proliferative efficacy induced by folate-conjugated zein/soy lecithin/carboxymethyl chitosan core−shell nanoparticles surpassed that of docetaxel solution, especially at higher drug concentrations, in both MCF-7 and SKOV-3 cells [54].

The potential application of Tf-bearing zein-based hybrid lipid nanoparticles for gene therapy was also examined. These nanoparticles exhibited effective DNA condensation, achieving over 80% condensation levels at polymer–DNA weight ratios of 1500:1 and 2000:1. This DNA condensation remained stable for a minimum of 24 h. While various studies have demonstrated that zein-based delivery systems can condense DNA, quantification of the condensation level has not always been provided. For instance, zein/sodium alginate nanocomposites have been shown to condense DNA using electrophoresis [63]. In another investigation, the DNA condensation by zein-oligochitosan-modified zein was shown to increase with an increasing amount of vector. Moreover, no degradation of plasmid DNA was observed for this polyplex [64].

Interestingly, the size of transferrin-bearing zein-based hybrid nanoparticles complexed with DNA was surprisingly high, consistently ranging from 400 nm to 590 nm. This was observed even at high polymer–DNA ratios of 2000:1 and 1500:1 that led to high DNA condensation. This was unexpected, particularly given that the size of control nanoparticles remained stable and below 400 nm after DNA complexation, and will require further investigation for clarification.

Furthermore, transferrin-bearing hybrid lipid nanoparticles complexed with DNA were bearing negative surface charges, closely resembling those observed with the Tf-bearing formulation entrapping docetaxel.

Despite the observed challenges related to nanoparticle size, and in line with outcomes of DNA condensation observed at the various ratios, transferrin-bearing zein-based hybrid lipid nanoparticles complexed to a plasmid DNA encoding β-galactosidase exhibited increased gene expression at optimal polymer–DNA ratios across the three tested cell lines, in comparison to DAB–DNA and PEI–DNA complexes. The highest level of transfection was achieved at a polymer–DNA weight ratio of 2000:1, which correlated with the highest degree of DNA condensation. It was up to 11-fold higher than that observed with DAB-DNA and PEI-DNA in LNCaP cells. This remarkable improvement surpassed the findings with zein/sodium alginate nanocomposites compared to PEI-DNA transfection [63]. Similarly, Kumari and colleagues reported that increasing the weight ratio could significantly enhance cell transfection efficiency for zein-oligochitosan polyplexes [64]. This polyplex exhibited superior gene transfection levels compared to TOOLSmooth-Fect and 293EZ-FECT transfection reagents. However, differences in cell lines and positive controls used in the two studies prevent direct comparison of the outcomes.

## 5. Conclusions

In this study, hybrid lipid nanoparticles composed of biocompatible and biodegradable zein, and conjugated to transferrin, were successfully prepared using the nanoprecipitation method. They effectively entrapped the hydrophobic drug docetaxel and demonstrated sustained release over a 24 h period, Notably, they exhibited a cumulative drug release of 72% at pH 5.5, mimicking the acidic endosomal pH of cancer cells. Furthermore, they exhibited enhanced cellular uptake of the drug, with an increase of up to 1.8-fold compared with the control formulation and the drug solution on PC-3 and LNCaP cancer cell lines. This heightened uptake was facilitated through mechanisms including caveolae-mediated endocytosis, which is an essential process for transferrin receptor-mediated endocytosis, and macropinocytosis. Treatment of PC-3 cells with these nanoparticles yielded a substantial increase in anti-proliferative efficacy when compared to docetaxel solution, at high docetaxel concentrations exceeding 1 µg/mL.

In addition, the targeted delivery system demonstrated the ability to condense DNA effectively, achieving condensation rates exceeding 80% at polymer–DNA weight ratios of 1500:1 and 2000:1. This led to an increased gene expression at optimal polymer–DNA ratios across all the three tested cell lines, in comparison to DA–DNA and PEI–DNA complexes. The highest level of transfection was up to 11-fold higher than that observed with DAB-DNA and PEI-DNA in LNCaP cells.

These novel transferrin-bearing zein-based hybrid lipid nanoparticles therefore hold promising prospects as a novel approach for prostate cancer treatment and warrant further investigation to assess the combination of docetaxel and plasmid DNA for cancer therapy.

## Figures and Tables

**Table 1:** Optimization of zein-based hybrid lipid nanoparticles. Values in green indicate the best results obtained.

	Size (nm)	Polydispersity Index (PDI)	Zeta Potential (mV)	Entrapment Efficiency (%)
Ethanol concentration (%)				
70	140.3 ± 0.8	0.06 ± 0.02	−29.5 ± 0.5	
80	146.6 ± 0.9	0.08 ± 0.01	−30.4 ± 0.8	
90	160.5 ±1.2	0.07 ± 0.01	−30.5 ± 1.0	
Preparation method				
Nanoprecipitation	205.7 ± 9.4	0.35 ± 0.06	−24.6 ± 0.8	74.8 ± 1.1
Coacervation	572.6 ± 37.5	0.19 ± 0.053	−21.2 ± 0.5	76.4 ± 2.8
Water: ethanol ratio (*v*/*v*)				
2:1	224.3 ± 28.0	0.27 ± 0.06	−21.8 ± 1.0	94.1 ± 2.7
3:1	217.1 ± 69.2	0.40 ± 0.06	−23.2 ± 0.8	51.9 ± 0.9
4:1	408 ± 73.1	0.67 ± 0.07	−23.1 ± 1.2	37.7 ± 3.0
5:1	178.9 ± 24.2	0.58 ± 0.09	−21.6 ± 0.9	32.0 ± 1.6
Use of probe sonication	236.4 ± 20.1	0.14 ± 0.05	−23.5 ± 3.4	
One-hour incubation	260.2 ± 3.1	0.38 ± 0.07	−20.9 ± 1.5	
Lipid: zein ratio (*w/w*)				
1:4	231.1 ± 3.4	0.17 ± 0.02	− 17.2 ± 0.7	87.9 ± 2.7
1:5	236.4 ± 20.1	0.14 ± 0.05	−23.5 ± 3.4	94.1 ± 2.7
1:6	224.8 ± 7.9	0.15 ± 0.03	−20.1 ± 0.7	89.9 ± 3.3
Zein amount (mg)				
30	236.4 ± 20.1	0.14 ± 0.05	−23.5 ± 3.4	80 ± 1.9
40	215.9 ± 6.8	0.16 ± 0.04	−18.4 ± 0.5	92.0 ± 0.9
50	225.3 ± 6.2	0.15 ± 0.01	−16.6 ± 0.0	83.7 ± 1.8
Amount of drug added related to zein weight (%)				
0.1	201.2 ± 6.7	0.11 ± 0.02	−17.2 ± 0.8	10.0 ± 0.2
0.5	215.9 ± 6.8	0.16 ± 0.04	−18.4 ± 0.5	92.0 ± 0.9

**Table 2:** Anti-proliferative efficacy of docetaxel entrapped in Tf-bearing and control zein-based hybrid lipid nanoparticles, or in solution, in PC3-Luc, DU145, and LNCaP cells. Results are expressed as mean ± S.E.M (*n* = 15).

IC_50_ (µg/mL) (mean ± S.E.M.)			
Formulations			
Cell Line	Tf-bearing LNP	Control LNP	Docetaxel Solution
PC3-Luc	0.25 ± 0.04	0.08 ± 0.02	0.0006 ± 8 × 10^−4^
DU145	0.02 ± 0.01	1.96 ± 0.54	0.0045 ± 0.0009
LNCaP	0.02 ± 0.00	0.05 ± 0.01	0.0023 ± 0.0014

## Data Availability

The data that support the findings of this study are available from the corresponding author, [CD], upon request.

## References

[1] Rawla P. (2019). Epidemiology of Prostate Cancer. World J. Oncol..

[2] Sung H., Ferlay J., Siegel R.L., Laversanne M., Soerjomataram I., Jemal A., Bray F. (2021). Global Cancer Statistics 2020: GLOBOCAN, Estimates of Incidence and Mortality Worldwide for 36 Cancers in 185 Countries. CA Cancer J. Clin..

[3] Wang L., Lu B., He M., Wang Y., Wang Z., Du L. (2022). Prostate Cancer Incidence and Mortality: Global Status and Temporal Trends in 89 Countries From 2000 to 2019. Front. Public Health.

[4] Siegel R.L., Miller D.M., Wagle N.S., Jemal A. (2023). Cancer statistics 2023. CA Cancer J. Clin..

[5] Zhao J., Guercio B.J., Sahasrabudhe D. (2023). Current Trends in Chemotherapy in the Treatment of Metastatic Prostate Cancer. Cancers.

[6] Luo D., Saltzman W.M. (2000). Synthetic DNA delivery systems. Nat. Biotechnol..

[7] Daniels T.R., Bernabeu E., Rodríguez J.A., Patel S., Kozman M., Chiappetta D.A., Holler E., Ljubimova J.Y., Helguera G., Penichet M.L. (2012). Transferrin receptors and the targeted delivery of therapeutic agents against cancer. Biochim. Biophys. Acta..

[8] Dufès C., Al Robaian M., Somani S. (2013). Transferrin and the transferrin receptor for the targeted delivery of therapeutic agents to the brain and cancer cells. Ther. Deliv..

[9] Al Robaian M., Chiam K.Y., Blatchford D.R., Dufès C. (2014). Therapeutic efficacy of intravenously administered transferrin-conjugated dendriplexes encoding TNF-α, TRAIL and interleukin-12 on prostate carcinomas. Nanomedicine.

[10] Shukla R., Cheryan M. (2001). Zein: The industrial protein from corn. Ind. Crop. Prod..

[11] Lawton J.W. (2002). Zein: A History of Processing and Use. Cereal Chem..

[12] Lin T., Lu C., Zhu L., Lu T. (2011). The Biodegradation of Zein In Vitro and In Vivo and its Application in Implants. AAPS PharmSciTech.

[13] Paliwal R., Palakurthi S. (2014). Zein in controlled drug delivery and tissue engineering. J. Control. Release.

[14] Gianazza E., Viglienghi V., Righetti P.G., Salamini F., Soave C. (1977). Amino acid composition of zein molecular components. Phytochemistry.

[15] Parris N., Cooke P.H., Hicks K.B. (2005). Encapsulation of Essential Oils in Zein Nanospherical Particles. J. Agric. Food Chem..

[16] Regier M.C., Taylor J.D., Borcyk T., Yang Y., Pannier A.K. (2012). Fabrication and characterization of DNA-loaded zein nanospheres. J. Nanobiotechnol..

[17] Dong F., Dong X., Zhou L., Xiao H., Ho P.Y., Wong M.S., Wang Y. (2016). Doxorubicin-loaded biodegradable self-assembly zein nanoparticle and its anti-cancer effect: Preparation, *in vitro* evaluation, and cellular uptake. Colloids Surf. B. Biointerfaces.

[18] Thapa R.K., Nguyen H.T., Jeong J.H., Shin B.S., Ku S.K., Choi H.G., Yong C.S., Kim J.O. (2017). Synergistic anticancer activity of combined histone deacytylase and proteasomal inhibitor-loaded zein nanoparticles in metastatic prostate cancers. Nanomed. NBM..

[19] Wen P., Ke W., Dirisala A., Toh K., Tanaka M., Li J. (2023). Stealth and pseudo-stealth nanocarriers. Adv. Drug Deliv. Rev..

[20] Podaralla S., Averineni R., Alqahtani M., Perumal O. (2012). Synthesis of Novel Biodegradable Methoxy Poly(ethylene glycol)–Zein Micelles for Effective Delivery of Curcumin. Mol. Pharm..

[21] Song R., Zhou Y., Li Y., Yang Z., Li F., Huang Q., Shi T., Zhang G. (2015). Preparation and characterization of mPEG-g-α-zein biohybrid micelles as a nano-carrier. J. Appl. Polym. Sci..

[22] Meewan J., Somani S., Laskar P., Irving C., Mullin M., Woods S., Roberts C.W., Alzahrani A.R., Ferro V.A., McGill S. (2022). Limited impact of the protein corona on the cellular uptake of PEGylated zein micelles by melanoma cancer cells. Pharmaceutics.

[23] Meewan J., Somani S., Almowalad J., Laskar P., Mullin M., MacKenzie G., Khadke S., Perrie Y., Dufès C. (2022). Preparation of zein-based nanoparticles: Nanoprecipitation versus microfluidic-assisted manufacture, effects of PEGylation on nanoparticle characteristics and cellular uptake by melanoma cells. Int. J. Nanomed..

[24] Fernandes M.A., Eloy J.O., Luiz M.T., Junior S.L.R., Borges J.C., Fuente L.R., Luis C.O., Marchetti J.M., Santos-Martinez M.J., Chorilli M. (2021). Transferrin-functionalized liposomes for docetaxel delivery to prostate cancer cells. Colloids Surf. A Physicochem. Eng..

[25] Mia Y., Liua Y., Feng S.-S. (2011). Formulation of Docetaxel by folic acid-conjugated d-α-tocopheryl polyethyleneglycol succinate 2000 (Vitamin E TPGS2k) micelles for targeted and synergistic chemotherapy. Biomaterials.

[26] Tan Q., Liu X., Fu X., Li Q., Dou J., Zhai G. (2012). Current development in nanoformulations of docetaxel. Expert. Opin. Drug Deliv..

[27] Chen J., Wu Z., Ding W., Xiao C., Zhang Y., Gao S., Gao Y., Cai W. (2020). SREBP1 siRNA enhance the docetaxel effect based on a bone-cancer dual-targeting biomimetic nanosystem against bone metastatic castration-resistant prostate cancer. Theranostics.

[28] Hermanson G.T. (2013). Dendrimers and Dendrons. Bioconjugate Techniques.

[29] Lowry O.H., Rosebrough N.J., Farr A.L., Randall R.J. (1951). Protein measurement with the Folin phenol reagent. J. Biol. Chem..

[30] Dufès C., Schätzlein A.G., Tetley L., Gray A.I., Watson D.G., Olivier J.C., Couet W., Uchegbu I.F. (2000). Niosomes and Polymeric Chitosan Based Vesicles Bearing Transferrin and Glucose Ligands for Drug Targeting. Pharm. Res..

[31] Paraman I., Lamsal B.P. (2011). Recovery and characterization of α-zein from corn fermentation coproducts. J. Agric. Food Chem..

[32] Maeda H. (1992). The tumor blood vessel as an ideal target for macromolecular anticancer agents. J. Control. Release.

[33] Maeda H., Wu J., Sawa T., Matsumura Y., Hori K. (2000). Tumor vascular permeability and the EPR effect in macromolecular therapeutics: A review. J. Control. Release.

[34] Li J., Kataoka K. (2021). Chemo-physical Strategies to Advance the in Vivo Functionality of Targeted Nanomedicine: The Next Generation. J. Am. Chem. Soc..

[35] Loftsson T., Brewster M.E. (2010). Pharmaceutical applications of cyclodextrins: Basic science and product development. J. Pharm. Pharmacol..

[36] Hua S., de Matos M.B.C., Metselaar J.M., Storm G. (2018). Current trends and challenges in the clinical translation of nanoparticulate nanomedicines: Pathways for translational development and commercialization. Front. Pharmacol..

[37] Čeh B., Winterhalter M., Frederik P.M., Vallner J.J., Lasic D.D. (1997). Stealth® liposomes: From theory to product. Adv. Drug Deliv. Rev..

[38] Barenholz Y. (2012). Doxil^®^—The first FDA-approved nano-drug: Lessons learned. J. Control. Release.

[39] Sharma S., Parmar A., Kori S., Sandhir R. (2016). PLGA-based nanoparticles: A new paradigm in biomedical applications. TrAC Trends Anal. Chem..

[40] Suzuki T., Sato E., Matsuda T., Tada H., Unno K., Kato T. (1989). Preparation of zein microspheres conjugated with antitumor drugs available for selective cancer chemotherapy and development of a simple colorimetric determination of drugs in microspheres. Chem. Pharm. Bull..

[41] Liu X., Sun Q., Wang H., Zhang L., Wang J. (2005). Microspheres of corn protein, zein for an invermectin drug delivery system. Biomaterials.

[42] Qian Z.M., Li H., Sun H., Ho K. (2002). Targeted drug delivery via the transferrin receptor-mediated endocytosis pathway. Pharmacol. Rev..

[43] Sakpakdeejaroen I., Somani S., Laskar P., Irving C., Mullin M., Dufès C. (2020). Anti-tumor activity of intravenously administered plumbagin entrapped in targeted nanoparticles. J. Biomed. Nanotechnol..

[44] Zhang L., Chan J.M., Gu F.X., Rhee J.W., Wang A.Z., Radovic-Moreno A.F., Alexis F., Langer R., Farokhzad O.C. (2008). Self-Assembled Lipid−Polymer Hybrid Nanoparticles: A Robust Drug Delivery Platform. ACS Nano.

[45] Zhan X., Tran K.K., Shen H. (2012). Effect of the poly(ethylene glycol) (PEG) density on the access and uptake of particles by antigen-presenting cells (APCs) after subcutaneous administration. Mol. Pharm..

[46] Zhang S., Tang C., Yin C. (2015). Effects of poly(ethylene glycol) grafting density on the tumor targeting efficacy of nanoparticles with ligand modification. Drug Deliv..

[47] Stenzel M.H. (2013). Bioconjugation Using Thiols: Old Chemistry Rediscovered to Connect Polymers with Nature’s Building Blocks. ACS Macro Lett..

[48] Yuan F., Dellian M., Fukumura D., Leunig M., Berk D.A., Torchilin V.P., Jain R.K. (1995). Vascular permeability in a human tumor xenograft: Molecular size dependence and cutoff size. Cancer Res..

[49] Ernsting M.J., Murakami M., Roy A., Li S.D. (2013). Factors Controlling the Pharmacokinetics, Biodistribution and Intratumoral Penetration of Nanoparticles. J. Control. Release.

[50] Blanco E., Shen H., Ferrari M. (2015). Principles of nanoparticle design for overcoming biological barriers to drug delivery. Nat. Biotechnol..

[51] Bhattacharjee S. (2016). DLS and zeta potential—What they are and what they are not?. J. Control. Release.

[52] Yu X., Han N., Dong Z., Dang Y., Zhang Q., Hu W., Wang C., Du S., Lu Y. (2022). Combined Chemo−Immuno−Photothermal Therapy for Effective Cancer Treatment via an All-in-One and One-for-All Nanoplatform. ACS Appl. Mater. Interfaces.

[53] Xing Y., Li X., Cui W., Xue M., Quan Y., Guo X. (2022). Glucose-Modified Zein Nanoparticles Enhance Oral Delivery of Docetaxel. Pharmaceutics.

[54] Wu Z., Li J., Zhang X., Li Y., Wei D., Tang L., Deng S., Liu G. (2022). Rational Fabrication of Folate-Conjugated Zein/Soy Lecithin/Carboxymethyl Chitosan Core−Shell Nanoparticles for Delivery of Docetaxel. ACS Omega.

[55] Lee H.S., Kang N.W., Kim H., Kim D.H., Chae J.W., Lee W., Song G.Y., Cho C.W., Kim D.D., Lee J.Y. (2021). Chondroitin sulfate-hybridized zein nanoparticles for tumor-targeted delivery of docetaxel. Carbohydr. Polym..

[56] Fredenberg S., Wahlgren M., Reslow M., Axelsson A. (2011). The mechanisms of drug release in poly(lactic-co-glycolic acid)-based drug delivery systems—A review. Int. J. Pharm..

[57] Guo L., Zhang H., Wang F., Liu P., Wang Y., Xia G., Liu R., Li X., Yin H., Jiang H. (2015). Targeted multidrug-resistance reversal in tumor based on PEG-PLL-PLGA polymer nano drug delivery system. Int. J. Nanomed..

[58] Zheng Y., Yu B., Weecharangsan W., Piao L., Darby M., Mao Y., Koynova R., Yang X., Li H., Xu S. (2010). Transferrin-conjugated lipid-coated PLGA nanoparticles for targeted delivery of aromatase inhibitor 7α-APTADD to breast cancer cells. Int. J. Pharm..

[59] Sutradhar K.B., Amin M.L. (2014). Nanotechnology in Cancer Drug Delivery and Selective Targeting. ISRN Nanotechnol..

[60] Deng Z., Manz D.H., Torti S.V., Torti F.M. (2017). Iron-responsive element-binding protein 2 plays an essential role in regulating prostate cancer cell growth. Oncotarget.

[61] Gao H., Yang Z., Zhang S., Cao S., Shen S., Pang Z., Jiang X. (2013). Ligand modified nanoparticles increases cell uptake, alters endocytosis and elevates glioma distribution and internalization. Sci. Rep..

[62] Oh N., Park J.H. (2014). Endocytosis and exocytosis of nanoparticles in mammalian cells. Int. J. Nanomed..

[63] Chen Y., Liu C., Yang Z., Sun Y., Chen X., Liu L. (2022). Fabrication of zein-based hydrophilic nanoparticles for efficient gene delivery by layer-by-layer assembly. Int. J. Biol. Macromol..

[64] Kumari M., Liu C.H., Wu W.C. (2018). Protein moiety in oligochitosan modified vector regulates internalization mechanism and gene delivery: Polyplex characterization, intracellular trafficking and transfection. Carbohydr. Polym..

